# 3D‐Printed Titanium Trabecular Scaffolds with Sustained Release of Hypoxia‐Induced Exosomes for Dual‐Mimetic Bone Regeneration

**DOI:** 10.1002/advs.202500599

**Published:** 2025-05-11

**Authors:** Lincong Luo, Weihan Zheng, Jiaying Li, Tingting Chen, Wanting Xue, Tao Lin, Mingrui Liu, Zi Yan, Jiaxin Yang, Jiamin Li, Jiahao Pu, Yaobin Wu, Konghe Hu, Shiyu Li, Wenhua Huang

**Affiliations:** ^1^ Yue Bei People's Hospital Postdoctoral Innovation Practice Base Southern Medical University Guangzhou Guangdong 510515 China; ^2^ Guangdong Engineering Research Center for Translation of Medical 3D Printing Application Guangdong Provincial Key Laboratory of Digital Medicine and Biomechanics National Key Discipline of Human Anatomy School of Basic Medical Sciences Southern Medical University Guangzhou Guangdong 510515 China; ^3^ Guangdong Medical Innovation Platform for Translation of 3D Printing Application The Third Affiliated Hospital of Southern Medical University Southern Medical University Guangzhou Guangdong 510630 China; ^4^ School of Basic Medical Sciences Fujian Medical University Fuzhou Fujian 350108 China; ^5^ School of Basic Medicine Dali University Dali Yunnan 671003 China; ^6^ School of Basic Medical Sciences Guangdong Medical University Dongguan Guangdong 523808 China; ^7^ Department of Microbiology and Immunology College of Basic Medicine and Public Hygiene Jinan University Guangzhou Guangdong 510632 China

**Keywords:** 3D‐printed, angiogenesis, biomimetic trabecular scaffold, bone regeneration, exosomes

## Abstract

Current Ti‐6Al‐4V bone implants lack trabecular structure and pro‑angiogenic cues, both essential for regeneration. Herein, a dual biomimetic strategy is devised that integrates a 3D‐printed biomimetic trabecular porous Ti‐6Al‐4V scaffold (BTPS) with exosome‐loaded PEGDA/GelMA hydrogel microspheres (PGHExo) designed for sustained release. BTPS is designed using Voronoi algorithms and imaging data, and replicates the geometry and mechanical properties of natural bone. Hypoxia‐induced human umbilical vein endothelial cell (HUVEC) derived exosomes (HExo) are encapsulated in PGHExo microspheres via microfluidic technology, enabling controlled release of HExo, and anchored onto BTPS using polydopamine (pDA) modification (BTPS&pDA@PGHExo). BTPS exhibited an elastic modulus of ≈3.2 GPa and a permeability of 11.52 × 10^−8^ mm^2^, mimicking natural bone. In vitro assays demonstrated that BTPS&pDA@PGHExo significantly enhanced osteogenesis and angiogenesis. mRNA‐Seq analysis suggested that BTPS&pDA@PGHExo regulates osteogenic and angiogenic gene expression through the activation of pathways including MAPK, mTOR, HIF‐1, and VEGF. In vivo, BTPS&pDA@PGHExo improved bone volume, density, and neovascularization in a rabbit model. This dual biomimetic strategy offers a promising clinical solution, addressing the limitations of conventional Ti‐6Al‐4V scaffolds and providing an innovative approach for personalized bone defect repair.

## Graphical abstract

This study presents 3D‐printed Ti‐6Al‐4V trabecular scaffolds combined with hypoxia‐induced exosomes encapsulated in PEGDA/GelMA microspheres for sustained release. The scaffolds mimic the structure of natural bone, while the exosome delivery system enhances osteogenesis and angiogenesis, significantly promoting bone regeneration and offering a promising strategy for the repair of large bone defects.

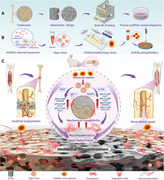

## 1 Introduction

Large segmental bone defects, arising from severe trauma, tumor resection, or infection, remain a pressing clinical challenge,^[^ ^1^ ^]^ frequently resulting in nonunion, implant failure, and prolonged morbidity.^[^ ^2^ ^]^ Conventional interventions, including autografts and allografts, are limited by donor scarcity and immunological risks,^[^ ^3^ ^]^ while synthetic ceramics and polymers^[^ ^4^ ^]^ often fail to provide the requisite mechanical strength and biological cues for robust, vascularized bone regeneration. Titanium alloys, particularly Ti‐6Al‐4V, have emerged as widely employed for load‐bearing implants due to their exceptional mechanical strength, corrosion resistance, and biocompatibility.^[^ ^5^ ^]^ Moreover, advances in additive manufacturing now permit patient‐specific implant designs with intricate porous architectures that closely mimic native bone structures.^[^ ^6^ ^]^ Yet, two critical barriers persist. First, the elastic modulus of Ti‐6Al‐4V (≈110 GPa) substantially exceeds that of natural bone (2–30 GPa), causing stress shielding and impaired osseointegration.^[^ ^7^ ^]^ Second, while mechanically superior, Ti‐6Al‐4V surfaces are intrinsically bioinert^[^ ^8^ ^]^ and lack the molecular signals essential for the formation of functional vascular networks that underpin sustained bone healing. Overcoming these dual challenges requires an integrated approach that marries mechanical optimization with dynamic biological signaling.

Nature's blueprint—the trabecular architecture of cancellous bone—offers both mechanical resilience and a porous network conducive to cellular infiltration and angiogenesis. Despite advances in computational modeling and 3D printing, replicating the inherent anisotropy and structural complexity of trabecular bone remains challenging. Most conventional scaffolds rely on regular geometric patterns that fail to capture this complexity, resulting in suboptimal stress distribution and limited osseointegration.^[^ ^9^ ^]^ By contrast, biomimetic trabecular porous structures inspired by native bone can reduce elastic modulus, mitigate stress shielding, and promote angiogenesis, thereby establishing a biomechanically and biologically favorable milieu for bone regeneration. Although these refined implants offer improved mechanical support and accelerated bone tissue formation, they often lack the endogenous cues necessary to drive robust vascularization and osteogenesis. Enhancing the bioactivity of biomimetic trabecular Ti‐6Al‐4V scaffolds is therefore crucial for achieving functional integration with native bone and advancing their clinical potential in large bone defect repair.

**Figure 1:**
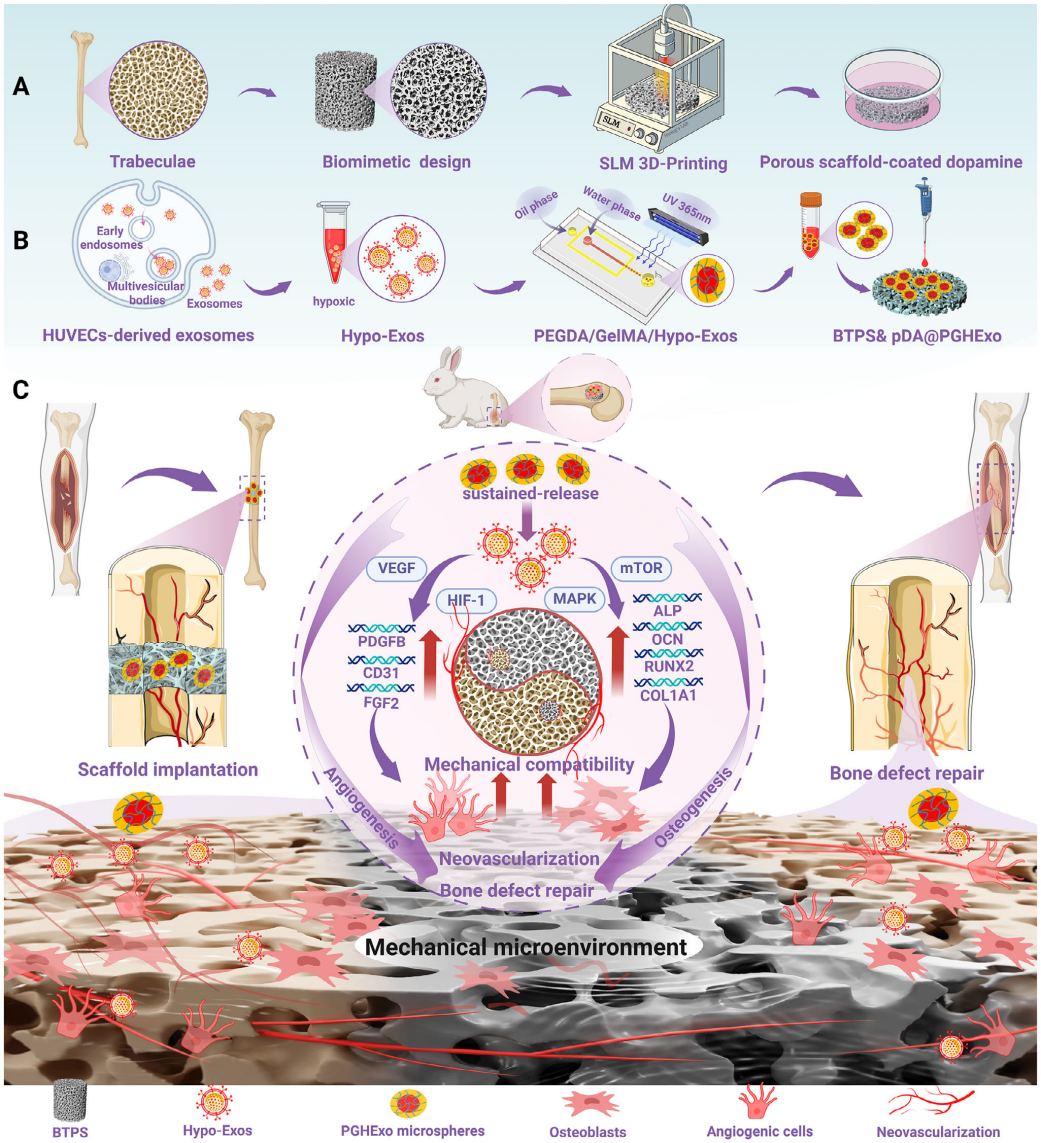
Schematic illustration of the fabrication process and therapeutic mechanism of BTPS&pDA@PGHExo scaffolds for large bone defect repair.

Among these indispensable biological cues, vascularization stands as a pivotal orchestrator, ensuring continuous nutrient and oxygen delivery, modulating local inflammation, and directing osteogenic cascades integral to sustained bone regeneration. Previous strategies—incorporating growth factors or pre‐seeding endothelial cells—have yielded incremental gains but often suffer from immunologic hurdles, low cell viability, and transient factor release.^[^ ^10^ ^]^ HUVEC‐derived exosomes, by contrast, offer stable, cell‐free delivery of pro‐angiogenic factors. Loaded with microRNAs, proteins, and lipids, exosomes can enhance endothelial sprouting, regulate local inflammation, and support osteoblast differentiation.^[^ ^11^ ^]^ Our preliminary studies indicate that sustained exosome delivery via microsphere‐based systems can be temporally synchronized with the vascularization phase of bone healing, providing extended therapeutic effects.^[^ ^12^ ^]^ Exosomes thus represent a scalable, cost‐effective, and biochemically stable modality for promoting vascularization in bone repair.^[^ ^13^ ^]^ However, the integration of soft exosomes into rigid Ti‐6Al‐4V scaffolds in a manner that preserves their bioactivity and spatial‐temporal release remains a significant challenge.

Here, we present a dual‐biomimetic strategy that seamlessly integrates a trabecular‐inspired Ti‐6Al‐4V scaffold with HUVEC‐derived exosomes encapsulated in dual‐network hydrogel microspheres engineered via advanced microfluidic techniques. By precisely tuning microsphere size and surface characteristics, we achieve sustained, controlled exosome release within the scaffold's pores, significantly enhancing exosome‐loading efficiency and prolonging bioactivity at the defect site. pDA modification establishes a robust adhesive interface,^[^ ^14^ ^]^ promoting stable exosome immobilization and ensuring compatibility within the biomimetic microenvironment. This localized bioactive reservoir continuously supplies exosomal factors, driving matrix formation and tissue integration at the bone interface.

Taken together, this study pioneers a dual‐biomimetic bone repair paradigm: a 3D‐printed, trabecular‐mimetic Ti‐6Al‐4V scaffold augmented with pDA‐anchored HExo microspheres for sustained exosome release. Critically, this composite design overcomes the conventional limitations of Ti‐6Al‐4V scaffolds—stress shielding and limited bioactivity—substantially enhancing osteogenesis and vascularized tissue regeneration. By integrating personalized treatment principles, our approach allows scaffolds to be tailored to patient‐specific needs, offering a safe, efficient, and adaptable platform for large bone defect repair. Through this synergy of structural and biological mimicry, we not only optimize scaffold mechanics but also achieve robust bioactivity, fostering superior vascularized bone regeneration and scaffold‐tissue integration. Ultimately, this work represents a significant advance in the clinical application of Ti‐6Al‐4V implants, underscoring its strong potential for translation into patient care. A schematic illustration of the scaffold fabrication and its therapeutic mechanism is shown in **Figure** 1.

## 2 Results

### 2.1 Design, Fabrication, and Characterization of BTPS

**Figure 2:**
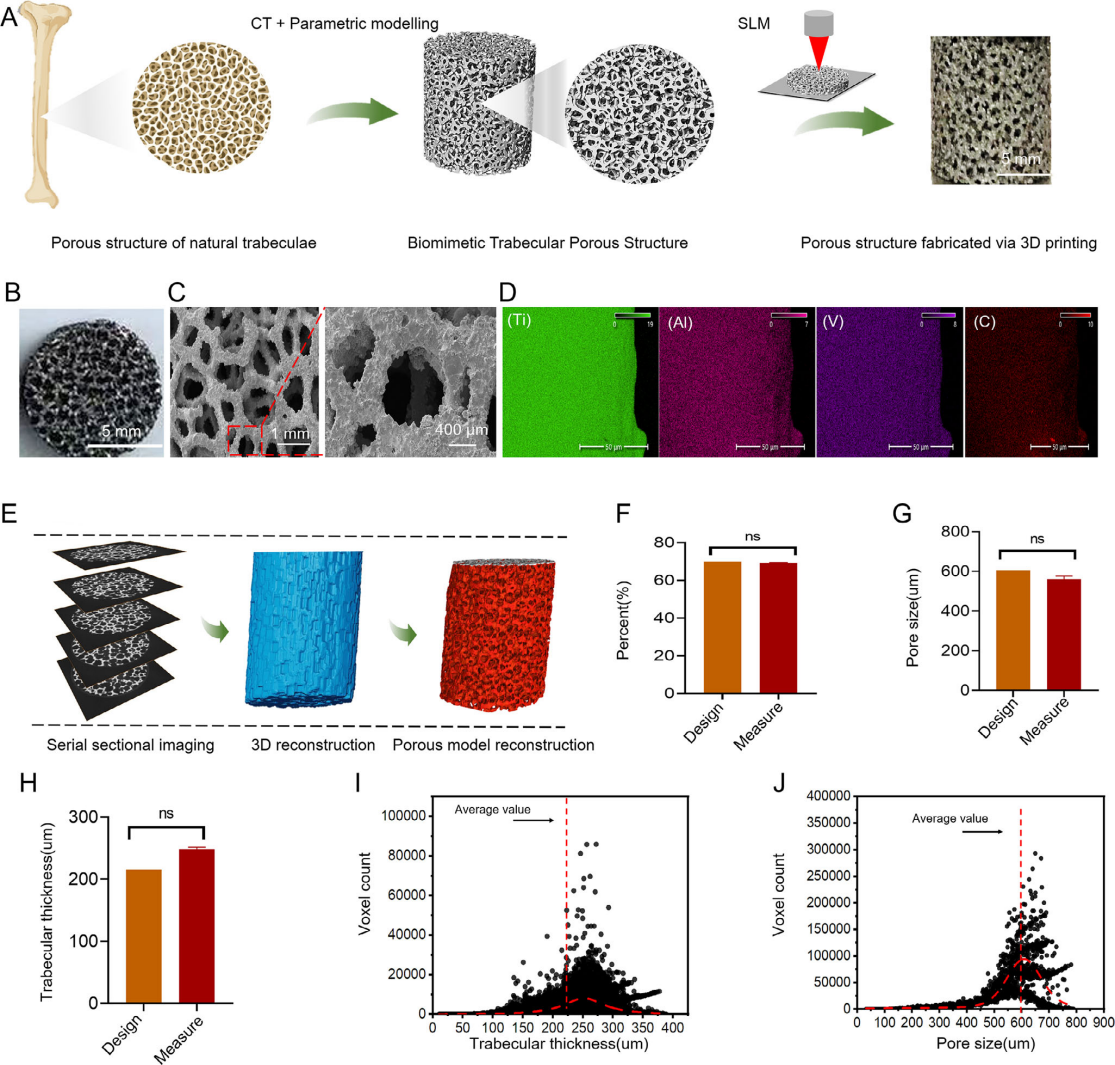
Characterization and elemental mapping of BTPS. A) Schematic illustration of the scaffold fabrication process, involving biomimetic trabecular design and SLM 3D printing. B) Macroscopic images display surface morphology of BTPS, scale bar, 5 mm. C) Scanning electron microscopy (SEM, Thermo Fisher, USA) images highlight microstructural differences, with scale bars at 400 µm and 1 mm. D) Elemental mapping via energy‐dispersive X‐ray spectroscopy (EDS, Thermo Fisher, USA) for BTPS, highlighting the distribution of key elements (Ti, C, Al, and V), with scale bar at 50 µm. E) Reconstruction process and quantitative analysis of scaffold microstructure. F) Comparison of designed and measured porosity (*n* = 3). G) Comparison of designed and measured pore sizes (*n* = 3). H) Comparison of designed and measured trabecular thickness (*n* = 3). I) Distribution of pore sizes with an average value marked by a red dashed line. J) Distribution of trabecular thickness with the average value indicated by a red dashed line. Data are presented as the mean ± standard deviation (Mean ± SD). Statistical analysis was performed using one‐way analysis of variance (ANOVA). ^∗^ *p* < 0.05, ns: *p* > 0.05.

We developed a BTPS that emulates the anatomical structure of the femoral cancellous bone (**Figure** **2**A). Through the Voronoi algorithm applied to imaging data, the resulting trabecular structure accurately mirrored the morphology and spatial organization of natural bone trabeculae. Following extensive parameter adjustments, an anisotropic BTPS was achieved with a 600 µm pore diameter and 70% porosity. This design demonstrated considerable geometric complexity and spatial distribution patterns, closely aligned with the structure of the femoral cancellous bone. The Ti‐6Al‐4V scaffold with a biomimetic porous trabecular design was successfully fabricated using Selective laser melting (SLM) technology.

The BTPS structure and composition were comprehensively evaluated using a range of advanced characterization techniques. As shown in SEM images (Figure 2B,C), the BTPS surfaces are smooth and free of microcracks or defects, with uniform pore wall thickness and a dense architecture. These characteristics reveal an anisotropic and interconnected porous structure. Moreover, the irregular pore network conforms well to design specifications. EDS analysis verified that the BTPS primarily comprises Titanium (Ti), Aluminum (Al), and Vanadium (V), aligning with Ti‐6Al‐4V standards, displaying uniform element distribution without impurities (Figure 2D). Furthermore, high‐resolution industrial computed tomography(CT) scanning verified the BTPS's internal integrity and connectivity, showing a porosity of 69.26%, closely matching the design target. Pore size analysis revealed an average pore size of 560 µm, predominantly centered ≈600 µm, and an average trabecular thickness of 248 µm, further confirming structural stability. Taken together, these findings align with the design objectives (Figure 2E–J), demonstrating that the BTPS achieves the intended structural precision and uniformity.

### 2.2 Finite Element, Mechanical, and Fluid Dynamics Analysis of BTPS

**Figure 3:**
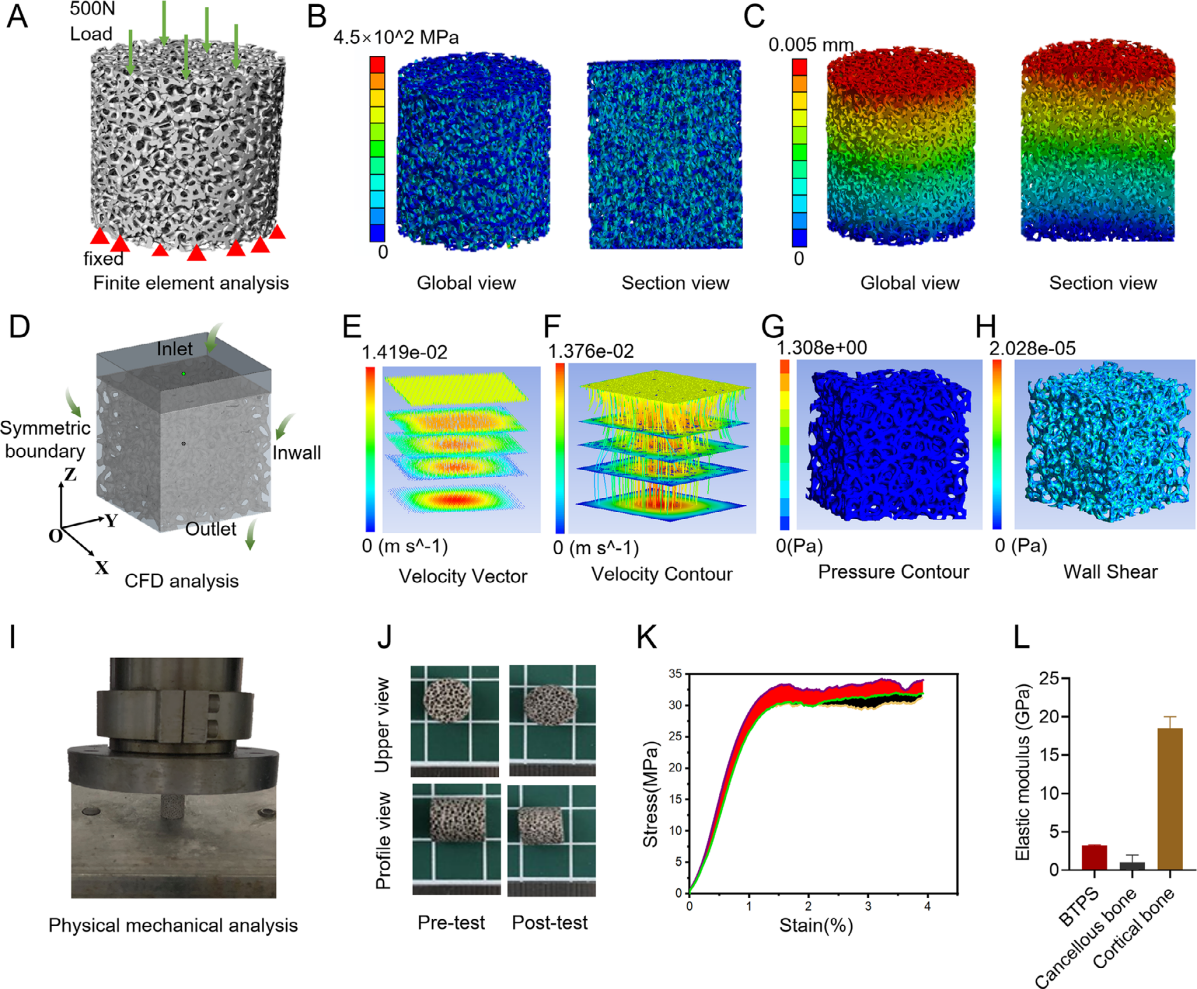
Mechanical characterization, finite element, and fluid dynamics analysis of BTPS. A) Finite element model of the BTPS under compressive loading, with boundary conditions applied. B) Von Mises stress distribution within the scaffold structure under compression. C) The global and section views show the distribution of displacement across the scaffold structure. D) Computational Fluid Dynamics (CFD) model setup with defined inlet, outlet, and symmetric boundary conditions. E) Velocity vector field illustrating flow direction and distribution within the scaffold. F) Velocity contour plot showing the magnitude of fluid flow across scaffold layers. G) Pressure contour illustrating pressure distribution throughout the scaffold. H) Wall shear stress contour indicating shear forces on scaffold surfaces. I) Compression testing setup for evaluating scaffold mechanical properties. J) Representative images of scaffolds before and after compression. K) Stress‐strain curve from compression testing, showing the load‐bearing capacity of BTPS (*n* = 3). L) Comparison of elastic modulus between BTPS, cancellous bone, and cortical bone (*n* = 3).

Finite element analysis (FEA) was conducted to assess the mechanical stability of the BTPS under applied loads (**Figure** **3**A–C). The design of the BTPS scaffold, featuring 600 µm pore size and 70% porosity, was selected based on literature and prior experimental data,^[^ ^15^, ^16^ ^]^ which support that this configuration strikes the optimal balance between mechanical performance, cellular infiltration, and nutrient exchange. This pore size and porosity combination was chosen for its ability to replicate the mechanical properties of natural bone while providing a biologically favorable environment for cell growth and nutrient flow, making it a representative scaffold for bone repair. The FEA focused on this optimized design. The analysis revealed a maximum stress of 454.26 MPa, indicating a uniform stress distribution without stress concentrations. The maximum displacement was 0.005 mm, localized primarily near the load application site, which suggests robust deformation resistance. Consistent with the results of physical mechanics experiments, these findings further validate the BTPS's mechanical stability and structural support capabilities.

Fluid mechanics analysis evaluated the BTPS's permeability and fluid transport properties (Figure 3D–H). Results reveal a uniform flow velocity within the BTPS, where fluid traverses the pore structure smoothly, without signs of turbulence or stagnation. The streamline diagram additionally clarifies the fluid pathway and consistent flow distribution, demonstrating the BTPS design's optimization of fluid channels. Shear force distributes evenly along the pore wall surface, with no excessive localized concentrations observed. Pressure distribution graphs reveal a notable gradient within the BTPS, particularly at the top and outlet, which signifies a substantial pressure differential. Such a gradient strongly suggests the structure's effective fluid channeling capacity. Permeability (K) measured at 11.52 × 10^−^⁸ confirms BTPS's high fluid transfer capacity, supporting its suitability for maintaining high permeability and mass transfer efficiency in biomechanical environments.

Physical mechanical testing demonstrated the BTPS's exceptional mechanical properties (Figure 3I–L). The stress‐strain curves were consistent, with BTPS showing an elastic modulus of 3.2 GPa, placing it squarely within the range of cancellous and cortical bone, thus closely replicating the mechanical properties of natural bone. Moreover, an average yield compression load of 3085.25 N indicates that BTPS provides the requisite mechanical strength necessary to support bone tissue repair and maintain structural integrity.

### 2.3 HUVECs‐Derived Exosomes Extraction and Characterization

**Figure 4:**
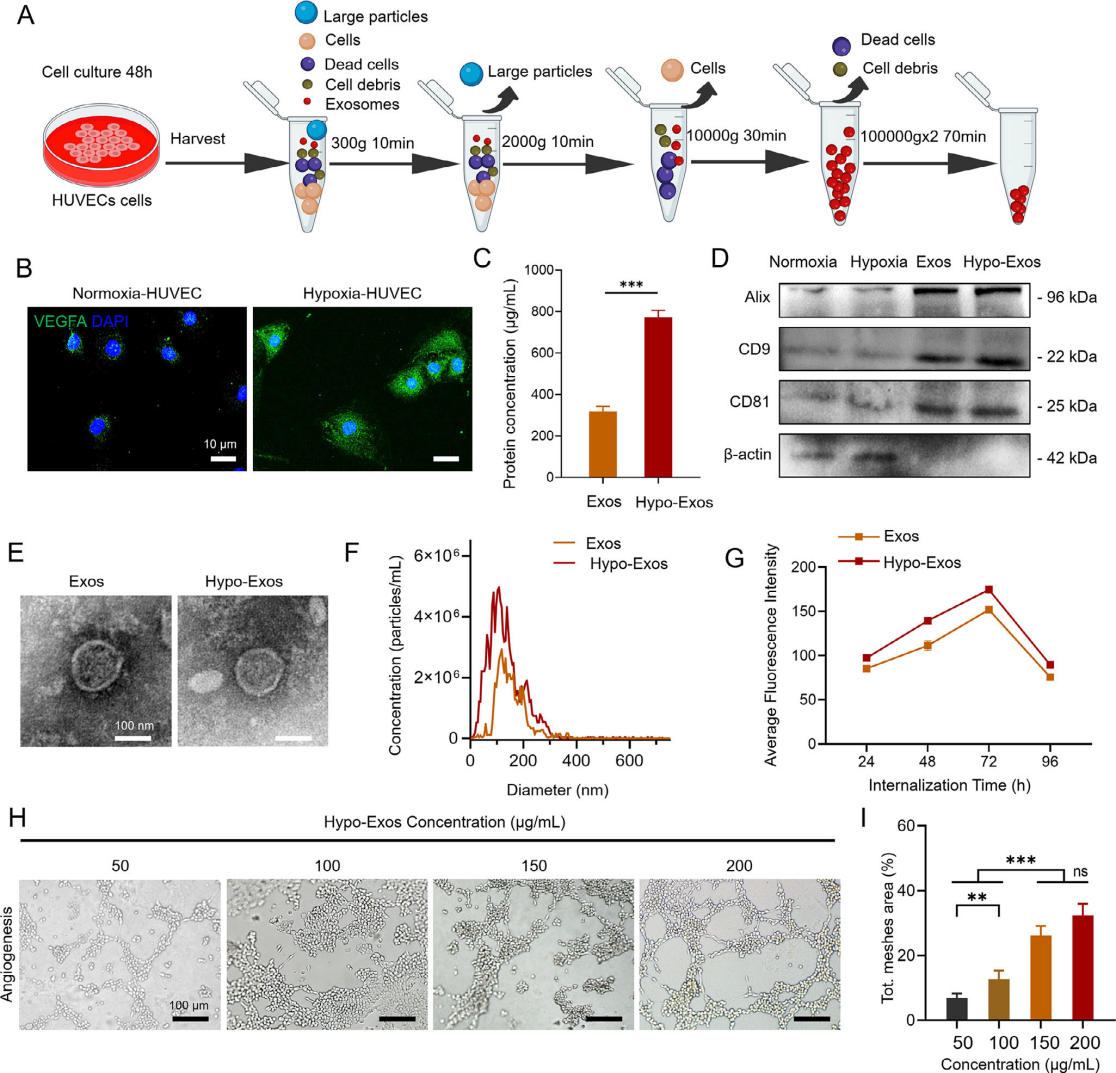
Isolation, characterization, and bioactivity evaluation of HUVEC ‐derived exosomes. A) Schematic representation of differential ultracentrifugation protocol employed for the isolation of exosomes. B) Immunofluorescence staining of VEGFA in HUVECs, visualizing VEGFA expression (green) with nuclear counterstaining (DAPI, blue), scale bar, 10 µm. C) Quantitative analysis of exosomal protein levels (*n* = 3). D) Western blot analysis of exosomal markers CD63, CD9, and Alix in purified exosomes, confirming exosome identity and integrity. E) TEM images of exosomes showing typical morphology and nanoscale size, scale bar, 100 nm. F) NTA of exosomes particle size distribution confirming homogeneity. G) Time‐dependent internalization of Exos and Hypo‐Exos by recipient cells (*n* = 3). H) Representative images showing angiogenic network formation at Hypo‐Exos concentrations of 50, 100, 150, and 200 µg mL^−1^, scale bar, 100 µm. I) Quantitative analysis of total mesh area across concentrations, indicating a concentration‐dependent increase in angiogenesis (*n* = 3). Data are presented as the Mean ± SD. Statistical analysis was performed using one‐way ANOVA. ns, no significant difference; ^**^ *p* < 0.01; and ^***^ *p* < 0.001.

As illustrated in **Figure** **4**A, HUVEC‐derived exosomes were successfully extracted under normoxic and hypoxic conditions via a multi‐step high‐speed centrifugation protocol. Immunofluorescence staining confirmed that hypoxia significantly elevated VEGFA expression in HUVECs (Figure 4B). Quantitative analysis of exosomal protein levels showed that Hypo‐Exos contained significantly higher protein concentrations than Exos (Figure 4C). Western blot analysis further validated the presence of established exosomal markers ALIX, CD9, and CD81 in both Exos and Hypo‐Exos, confirming the purity and identity of the exosomes (Figure 4D). Transmission Electron Microscopy (TEM) imaging revealed that both Exos and Hypo‐Exos exhibited a characteristic round morphology, uniform structure, and an approximate diameter of 100 nm (Figure 4E). Nanoparticle Tracking Analysis (NTA) results indicated a greater particle concentration in Hypo‐Exos compared to Exos, although both maintained similar particle size distributions predominantly between 100 and 150 nm (Figure 4F). Fluorescence microscopy analysis revealed a time‐dependent internalization of PKH26‐labeled Exos and Hypo‐Exos by recipient cells, with a significantly enhanced uptake of Hypo‐Exos observed at 72 h. The quantitative assessment further demonstrated a peak in exosome internalization at 72 h for both Exos and Hypo‐Exos, with Hypo‐Exos consistently exhibiting higher cellular uptake throughout the time points studied (Figure 4G; Figure S2, Supporting Information). Collectively, these findings suggest that Hypo‐Exos displays superior concentration and functional properties under hypoxic conditions compared to normoxia. Subsequently, the pro‐angiogenic potential of Hypo‐Exos was examined across various concentrations (50, 100, 150, and 200 µg mL^−1^). The findings revealed that increased Hypo‐Exos concentrations were associated with an upsurge in vascular‐like structures, clearly demonstrating a pro‐angiogenic effect. Notably, the 200 µg mL^−1^ Hypo‐Exos group exhibited the most prominent formation of vascular structures (Figure 4H). The quantitative analysis presented in the figure confirms a dose‐dependent effect of Hypo‐Exos within the range of 50–150 µg mL^−1^, showing pronounced angiogenesis with rising concentrations. At 200 µg mL^−1^, the pro‐angiogenic effect plateaued, suggesting a saturation threshold and an optimal concentration for angiogenesis (Figure 4I).

### 2.4 Preparation and Characterization of PGHExo Microspheres

**Figure 5:**
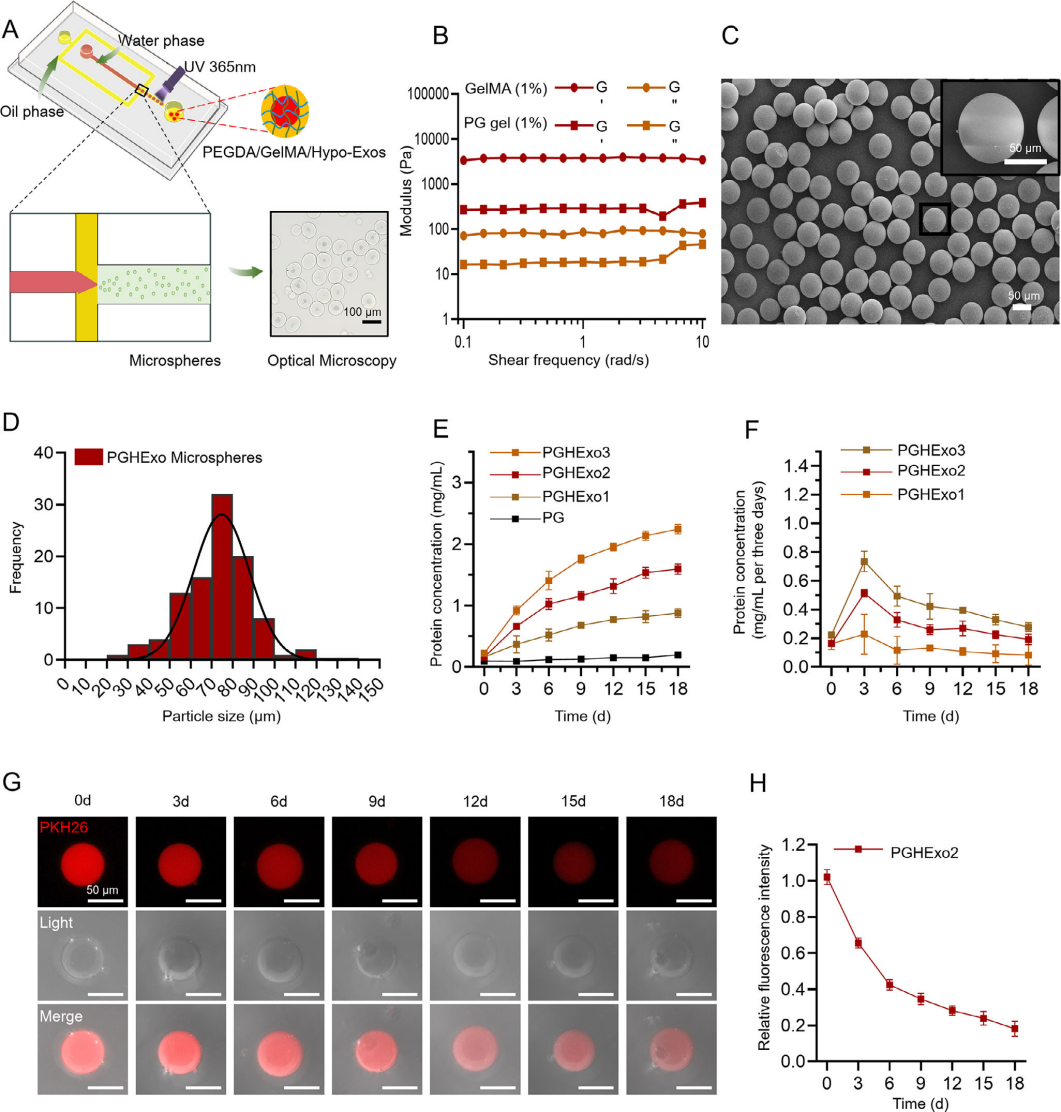
Fabrication, characterization, and release profile of PEGDA/GelMA dual‐network hydrogel sustained‐release microspheres loaded with Hypo‐Exos. A) Schematic of the microfluidic chip setup for generating uniform PGHExo, alongside a bright‐field microscopy image demonstrating high monodispersity, scale bar, 100 µm. B) Rheological properties of 1% GelMA and 1% PGgel (*n* = 3). C) SEM image revealing surface morphology and consistent size distribution of microspheres. The inset shows an enlarged view, scale bar, 50 µm. D) Histogram of microsphere size distribution. E) Cumulative protein release profile of PGHExo microspheres over 18 days (*n* = 3). F) Concentration of protein released every 3 days (*n* = 3). G) Confocal microscopy images of PKH26‐labeled PGHExo2 microspheres showing exosome release at different time points (0d, 3d, 6d, 9d, 12d, 15d, and 18d). Top row: fluorescence images; Middle row: light images; Bottom row: merged images. Scale bar, 50 µm. H) Quantitative analysis of relative fluorescence intensity over time, indicating a gradual decrease in fluorescence, reflecting sustained exosome release from PGHExo2 microspheres (*n* = 3).

**Figure** **5**A illustrates the successful fabrication of PGHExo dual‐network hydrogel sustained‐release microspheres via microfluidic chip technology. The microspheres exhibited smooth surfaces, homogeneous internal structures, and well‐defined morphology, with no apparent defects. Particle size distribution analysis revealed that the microspheres maintained a consistent size, averaging ≈80 µm within a range of 60–90 µm(Figure 5C,D). Rheological analysis indicated that the PEGDA/GelMA (PG) gel sustained a stable storage modulus (G*′*) and loss modulus (G″) across various shear frequencies, exceeding those of the GelMA single‐component gel, thereby demonstrating superior mechanical stability (Figure 5B). To evaluate protein release profiles, PG samples with Hypo‐Exos at various concentrations (1‐3 mg mL^−1^; PGHExo1, PGHExo2, and PGHExo3) and without Hypo‐Exos were quantified. Notably, PG samples lacking Hypo‐Exos exhibited minimal protein release throughout the 18‐day period. Conversely, samples containing Hypo‐Exos demonstrated sustained protein release, with release rates directly correlated to Hypo‐Exo concentration (Figure 5E). Given that the peak internalization of exosomes occurred at 72 h, we monitored the release concentration of PGHExo2 every three days. The average release concentration remained ≈200 µg mL^−1^ (Figure 5F), which is optimal for angiogenesis induction. These findings confirm PGHExo2 as the most promising candidate for bone repair applications. To monitor PKH26‐labeled Hypo‐Exos release, confocal microscopy was employed. The observations revealed that Hypo‐Exos in PGHExo2 were gradually released, sustaining for up to 18 days (Figure 5G). Both immunofluorescence imaging and quantitative fluorescence analysis of PGHExo2 revealed consistent trends, confirming effective Hypo‐Exo release within the dual‐network microspheres (Figure 5H). Collectively, these findings underscore that PGHExo dual‐network microspheres exhibit favorable stability and controlled‐release capabilities, supporting their suitability for sustained exosome delivery to HUVECs and enhanced scaffold bioactivity.

### 2.5 In Vitro Bioactivity Assessment of BTPS&pDA@PGHExo

**Figure 6:**
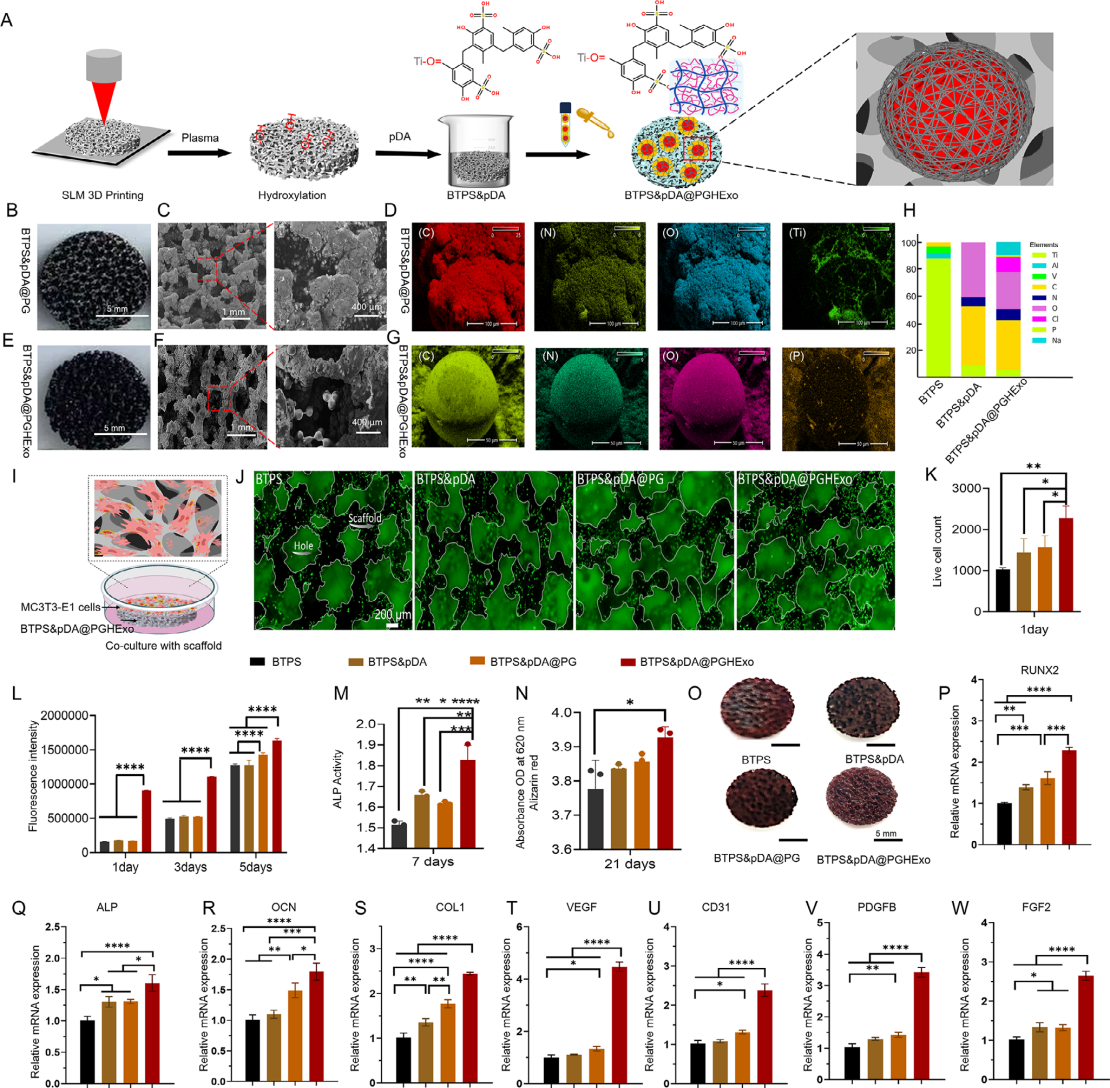
Development, characterization, and functional assessment of BTPS&pDA@PGHExo. A) Schematic illustrating the fabrication of the BTPS&pDA@PGHExo. B) Macroscopic images display surface morphology of BTPS&pDA, scale bar, 5 mm. C) SEM images of BTPS&pDA, with scale bars at 400 µm and 1 mm. D) Elemental mapping via EDS for BTPS&pDA, highlighting the distribution of key elements (C, N, O, and Ti), with scale bar at 100 µm. E) Macroscopic images display surface morphology of BTPS&pDA@PGHExo, scale bar, 5 mm. F) SEM images of BTPS&pDA@PGHExo, with scale bars at 400 µm and 1 mm. G) Elemental mapping via EDS for BTPS&pDA@PGHExo, highlighting the distribution of key elements (C, N, O, and P) and (Ti, Cl, and Na) (Figure S3, Supporting Information), with scale bar at 50 µm. H) Quantitative analysis of elemental composition, demonstrating uniform distribution and effective surface modification, confirming the successful integration of PGHExo microspheres within the scaffold structure. I) Schematic of MC3T3‐E1 cell co‐culture with each scaffold group. J) Live cell fluorescence imaging on BTPS, BTPS&pDA, BTPS&pDA@PG, and BTPS&pDA@PGHExo scaffolds. Scale bar, 200 µm. K) Quantification of live cell count on different scaffolds (*n* = 3). L) Fluorescence intensity measurements over 1, 3, and 5 days, indicated increased cell proliferation on modified scaffolds, particularly on BTPS&pDA@PG (*n* = 3). M) Alkaline Phosphatase (ALP) activity at day 7, showing elevated osteogenic differentiation on BTPS&pDA@PG scaffolds (*n* = 3). N) Quantitative analysis of mineralization at 21 days, with absorbance measured at 620 nm, indicated significantly higher mineralization on BTPS&pDA@PGHExo (*n* = 3). O) Macroscopic images of BTPS, BTPS&pDA, BTPS&pDA@PG, and BTPS&pDA@PGHExo after Alizarin Red staining. Scale bar, 5 mm. P–S) The Real‐time PCR (RT‐PCR)results of osteogenesis‐associated gene expression (*n* = 3). T–W) The RT‐PCR results of angiogenesis‐associated gene expression (*n* = 3). Figures 6K–N,P–W share a single set of grouping information. Data are presented as the Mean ± SD. Statistical analysis was performed using one‐way ANOVA. ^*^ *p* < 0.05; ^**^ *p* < 0.01; ^***^ *p* < 0.001; and ^****^ *p* < 0.0001.

We initiated the fabrication and performed a detailed characterization of BTPS&pDA@PGHExo. **Figure** **6**A illustrates the fabrication process and the chemical interactions between the 3D‐printed BTPS scaffold, the PDA coating, and the externally loaded PGHExo. The BTPS scaffold underwent plasma treatment to introduce hydroxyl (─OH) groups, which facilitate the adhesion of the PDA coating. This coating, in turn, promotes the attachment of the PGHExo microspheres, ensuring that the exosome‐loaded microspheres are securely anchored to the scaffold, forming an integrated structure. The untreated BTPS presented a silvery‐white appearance upon visual inspection (Figure 2B). After pDA modification, the BTPS surface was uniformly coated with a black film (Figure 6B). SEM imaging confirmed the presence of a continuous pDA coating on the BTPS surface (Figure 6C). EDS analysis (Figure 6D) indicated that BTPS&pDA was primarily composed of carbon (C), nitrogen (N), oxygen (O), and Ti, thereby validating successful pDA coating. SEM images of BTPS&pDA@PGHExo showed a uniform distribution of PGHExo microspheres on the BTPS surface, suggesting favorable adhesion properties. Elemental analysis further identified C, O, N, Ti, phosphorus (P), sodium (Na), and chloride (Cl) (Figure 6E–H; Figure S3, Supporting Information). Notably, the detected C, O, N, and P within the exosomes likely originated from their complex composition, comprising lipids, proteins, and RNA. The detected Na and Cl likely resulted from saline treatment. Together, these findings confirm the successful pDA coating modification and efficient assembly of PGHExo microspheres onto the BTPS surface.

Cell viability assays demonstrated that all scaffold materials exhibited strong cytocompatibility, showing no signs of cell death. Importantly, MC3T3‐E1 cell survival rates in the BTPS&pDA@PGHExo group were significantly elevated compared to other groups, as evidenced by an intensified green fluorescence signal. Qualitative results corroborated the increased cell viability and survival rates in this group, underscoring the scaffold's potential to promote cell survival and functional maintenance (Figure 6I–K). Cell proliferation assays further demonstrated the superior biocompatibility of the BTPS&pDA@PGHExo. Cell proliferation rates increased progressively over time across all samples (Figure 6L). The BTPS&pDA@PGHExo exhibited significantly higher fluorescence intensity, indicating the most robust cell proliferation among the groups. In contrast, the BTPS and BTPS&pDA groups showed comparatively lower proliferation rates, while the BTPS&pDA@PG group displayed a moderate increase. These findings are consistent with the live/dead cell data, reinforcing the enhanced capacity of the BTPS&pDA@PGHExo to support cell growth.

The ALP assay and Alizarin Red S mineralization assay were performed to evaluate the osteogenic potential of the scaffolds. The BTPS&pDA@PGHExo group exhibited the highest ALP activity among all groups, significantly surpassing the BTPS, BTPS&pDA, and BTPS&pDA@PG groups, indicating an enhanced osteogenic differentiation (Figure 6M). After 21 days of incubation in an osteogenic medium, the BTPS&pDA@PGHExo also demonstrated substantial mineralized nodule formation, evidenced by more intense reddish‐orange staining compared to other groups (Figure 6O). Quantitative analysis confirmed that this group achieved the highest mineralization level, with absorbance values significantly higher than those of the other groups (Figure 6N). RT‐PCR analysis further supported these findings by showing a significant upregulation of osteogenesis‐related markers—including ALP, OCN, COL1, and RUNX2—in the BTPS&pDA@PGHExo group compared to the other groups (Figure 6P–S). This group consistently demonstrated the highest levels of osteogenic gene expression, indicating the synergistic effects of the exosome incorporation. These results suggest that the BTPS&pDA@PGHExo significantly promotes osteogenic differentiation and mineralization, potentially by enhancing the mineralization conditions within the extracellular matrix.

Beyond its osteogenic properties, the BTPS&pDA@PGHExo scaffold demonstrated significant pro‐angiogenic potential. Angiogenesis was assessed by quantifying mRNA expression levels of VEGF, CD31, PDGFB, and FGF2. Among the groups, BTPS&pDA@PGHExo exhibited the highest expression levels for all markers, with VEGF showing a nearly sixfold upregulation (*p* < 0.0001). Similarly, CD31, PDGFB, and FGF2 were significantly upregulated (*p* < 0.0001), indicating enhanced angiogenic activity. These findings underscore the exosome‐enriched scaffold's ability to establish a pro‐angiogenic microenvironment, which is critical for effective bone regeneration (Figure 6T–W).

### 2.6 mRNA‐seq Analyses of BTPS&pDA@PGHExo

**Figure 7:**
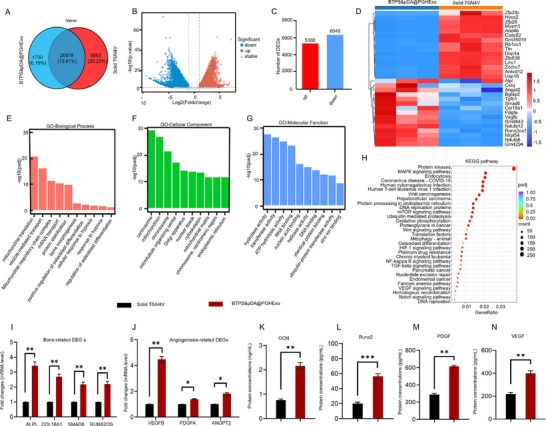
mRNA‐seq analyses of gene expression. A) Venn diagram showing the overlap of DEGs between two scaffold treatment groups. B) Volcano plot showing DEGs between scaffold treatment groups, with upregulated (red) and downregulated (blue) genes highlighted. C) Bar chart illustrating the total number of upregulated and downregulated DEGs. D) Heatmap of DEGs between BTPS&pDA@PGHExo and Solid Ti‐6Al‐4V scaffold treatments, with red indicating upregulated genes and blue indicating downregulated genes. E–G) GO enrichment analysis of DEGs categorized by Biological Process (E), Cellular Component (F), and Molecular Function (G). H) KEGG pathway enrichment analysis of DEGs, identifying significantly enriched pathways such as MAPK, mTOR, HIF‐1, and VEGF signaling, which are associated with scaffold‐induced cellular responses. The size and color of each dot represent the count of DEGs in each pathway and the adjusted p‐value, respectively. I) RT‐PCR analysis of bone‐related DEGs (*n* = 3). J) RT‐PCR analysis of angiogenesis‐related DEGs (*n* = 3). K–N) Enzyme‐Linked Immunosorbent Assay (ELISA) quantification of protein levels: The protein concentrations of RUNX2, OCN, PDGF, and VEGF were measured by ELISA (*n* = 3). Figure 7I–N share a single set of grouping information. Data are presented as the Mean ± SD. Statistical analysis was performed using one‐way ANOVA. ^*^ *p* < 0.05; ^**^ *p* < 0.01; and ^***^ *p* < 0.001.

The mRNA‐seq analyses revealed a total of 28991 genes, with 20678 (73.61%) commonly expressed between the two groups. The volcano plot demonstrated significant transcriptional changes, including 5102 upregulated and 6049 downregulated genes. Gene Ontology (GO)enrichment analysis identified key biological processes such as mitochondrial function, bone morphogenesis, and hypoxia‐induced angiogenesis, alongside cellular components and molecular functions like extracellular matrix organization and growth factor binding. Additionally, Kyoto Encyclopedia of Genes and Genomes (KEGG)pathway analysis highlighted critical signaling pathways, including MAPK, mTOR, HIF‐1, and VEGF, as pivotal regulators of osteogenesis and angiogenesis. The heatmap analysis of DEGs underscored significant genes, including ALPL, COL18A1, SAMD6, and RUNX2OS1 for bone‐related functions, as well as VEGFB, PDGFA, and ANGPT2 for angiogenesis. These findings demonstrate the BTPS&pDA@PGHExo ability to modulate key molecular pathways, orchestrate osteogenic and angiogenic responses, and facilitate extracellular matrix remodeling, further supporting its potential for enhanced bone regeneration and vascularisation in translational applications (**Figure** **7**A–H).

To validate the RNA‐seq findings, quantitative Real‐Time PCR(qRT‐PCR) was performed on key osteogenic and angiogenic DEGs (Figure 7I,J). The results showed a significant increase in ALPL, COL18A1, SAMD6, and RUNX2OS1 expression in the BTPS&pDA@PGHExo group compared to the control, confirming RNA‐seq‐predicted upregulation of bone‐related genes. Similarly, VEGFB, PDGFA, and ANGPT2 expression was significantly elevated, verifying the angiogenic potential. Furthermore, ELISA quantification confirmed increased protein expression levels of RUNX2, OCN, PDGF, and VEGF in the BTPS&pDA@PGHExo group (Figure 7K–N). These findings demonstrate that the transcriptional changes observed in RNA‐seq data are effectively translated into protein‐level expression, reinforcing the functional impact of BTPS&pDA@PGHExo on bone regeneration and angiogenesis.

### 2.7 In Vivo Bone Defect Repair with BTPS&pDA@PGHExo

**Figure 8:**
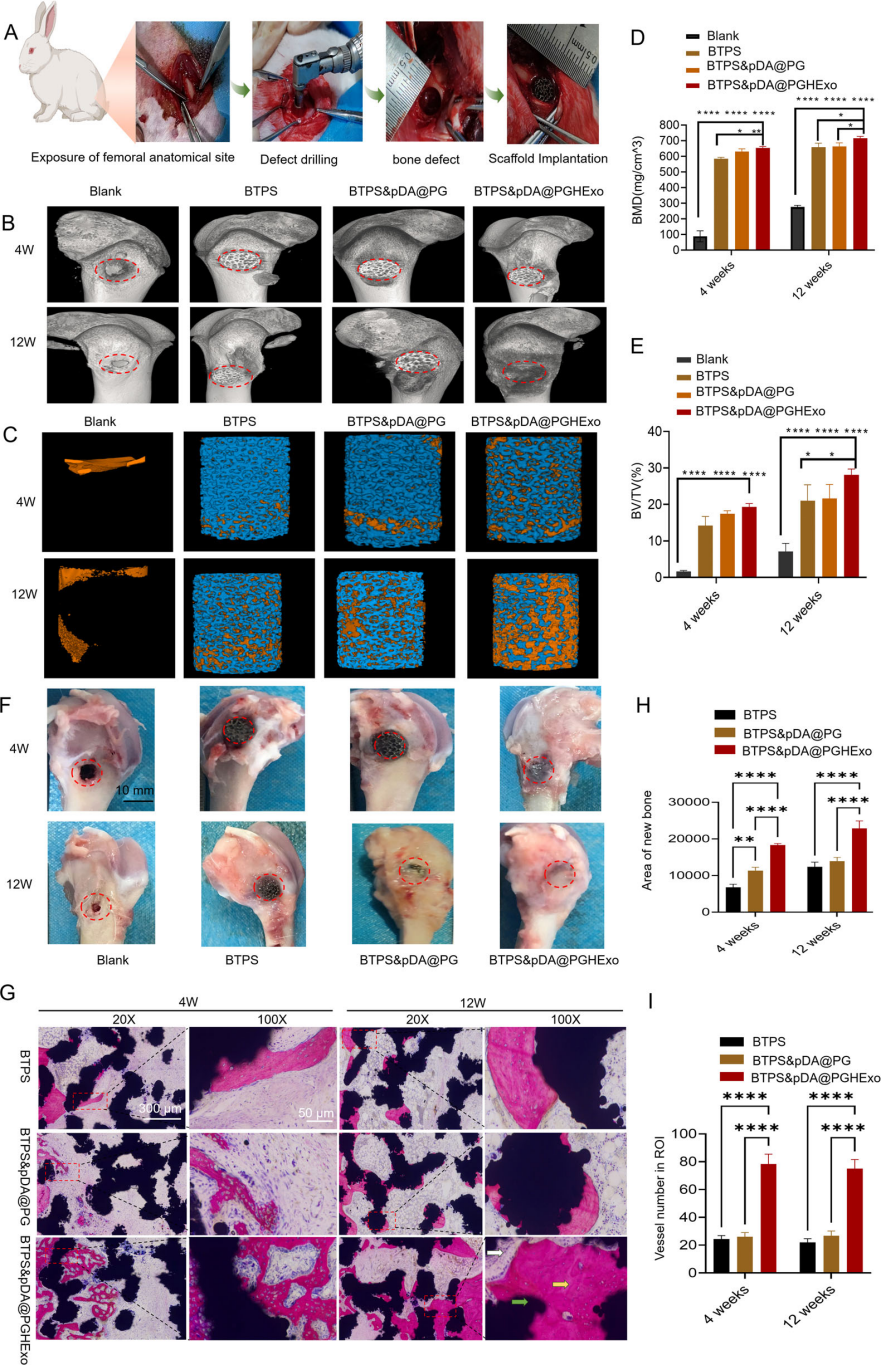
In vivo assessment of bone regeneration efficacy across scaffold groups in a rabbit femoral defect model. A) Schematic overview and intraoperative photographs illustrating the creation of femoral defects and scaffold implantation in a rabbit model. B) Representative Micro‐CT images showing defect sites treated with Blank, BTPS, BTPS&pDA@PG, and BTPS&pDA@PGHExo scaffolds, highlighting differences in new bone formation across groups. C) 3D Micro‐CT reconstructions and sectional views demonstrating scaffold integration and bone ingrowth within the defect regions for each scaffold type. D) Bone volume to total volume ratio (BV/TV%) analysis at 4 and 12 weeks post‐implantation, showed significantly higher bone formation in BTPS&pDA@PGHExo scaffolds compared to other groups (*n* = 3). E) Bone mineral density (BMD) analysis at 4 and 12 weeks, indicating enhanced mineralization in the BTPS&pDA@PGHExo scaffold group (*n* = 3). F) Representative macroscopic images of femoral defect sites at various time points post‐implantation, comparing healing progression among the Blank, BTPS, BTPS&pDA@PG, and BTPS&pDA@PGHExo groups. G) Methylene blue/fuchsin‐stained sections of BTPS, BTPS&pDA@PG, and BTPS&pDA@PGHExo scaffolds at 4 weeks (left panels) and 12 weeks (right panels) post‐implantation. Images captured at different magnifications highlight differences in new bone formation, cellular infiltration, and scaffold integration. Yellow arrows denote new bone formation, green arrows indicate scaffold material and white arrows point to newly formed blood vessels. Scale bars, 300 and 50 µm. H) Area of new bone formation for BTPS, BTPS&pDA@PG, and BTPS&pDA@PGHExo scaffolds, with BTPS&pDA@PGHExo showing significantly increased bone formation at both 4 and 12 weeks (*n* = 3). I) Vessel count in the region of interest (ROI) across scaffold groups, indicating enhanced neovascularization in the BTPS&pDA@PGHExo group (*n* = 3). Data are presented as the Mean ± SD. Statistical analysis was performed using one‐way ANOVA. ^*^ *p* < 0.05; ^**^ *p* < 0.01; and ^****^ *p* < 0.0001.

The efficacy of various implants in bone defect repair was comprehensively assessed using Microcomputed Tomography (Micro‐CT) and histopathological analyses at 4 and 12 weeks post‐surgery. **Figure** **8**A–E demonstrates that the BTPS&pDA@PGHExo group exhibited significantly higher new bone volume and density compared to other groups at both time points. Specifically, at 4 weeks, this group achieved new bone volumes of 19.3 mm^3^ and bone densities of 653.9 mg cm^−^^3^, which further increased to 28.1 mm^3^ and 717.1 mg cm^−^^3^ by 12 weeks (*p* < 0.05). In contrast, the BTPS and BTPS&pDA@PG groups showed diminished new bone formation and density, while the blank group presented the least favorable outcomes. The complementary histopathological analysis presented in Figure 8F–I revealed that the BTPS&pDA@PGHExo implants facilitated significant new bone formation and a denser vascular network within the defect site at both 4 and 12 weeks. At the early stage (4 weeks), there was notable new bone presence and vascularization, which progressed to advanced bone maturation and vascular densification by 12 weeks. Quantitative assessments confirmed that both new bone volume and vascular density were markedly elevated in the BTPS&pDA@PGHExo group compared to controls at both time points (*p* < 0.05). Additionally, histological examinations indicated more uniform cell morphology and a denser tissue structure in the BTPS&pDA@PGHExo group, underscoring the implant's enhanced osteogenic and angiogenic potential. Collectively, the integrated Micro‐CT and histopathological data illustrate that the BTPS&pDA@PGHExo composite scaffold significantly promotes bone regeneration and vascularization, demonstrating superior performance in facilitating bone defect repair relative to other tested implants.

## 3 Discussion

In this study, we introduce a dual biomimetic framework that intricately combines anatomical fidelity and mechanical congruence with sustained, biologically active signaling to enhance vascularized bone regeneration. Utilizing a data‐driven 3D‐printing methodology alongside a Voronoi‐based architectural design, our Ti‐6Al‐4V scaffold meticulously replicates the porous trabecular structure and mechanical properties of native cancellous bone, effectively mitigating stress shielding and facilitating cellular infiltration and tissue remodeling. Simultaneously, the incorporation of a PEGDA/GelMA dual‐network hydrogel microsphere enables the controlled, long‐term release of HExo, providing continuous pro‐osteogenic and pro‐angiogenic stimuli. This synergistic integration of structural and biochemical elements establishes a robust regenerative platform that promotes substantial new bone formation and a well‐organized vascular network, addressing significant challenges in orthopedic tissue engineering and offering a promising solution for the repair of large bone defects.

The microstructure and mechanical properties of bone tissue are essential in supporting effective bone regeneration.^[^ ^17^ ^]^ The integration of image data with a Voronoi algorithm facilitated the construction of a 3D porous structure that closely mimics natural bone trabeculae. This anatomically‐driven data provides a solid foundation for precise and tailored scaffold design. The Voronoi algorithm translated trabecular structural data into a mathematical model, enabling precise customization of pore geometry and spatial heterogeneity. This advanced approach closely replicates the mechanical and biological properties of natural bone, surpassing conventional isotropic designs by better addressing directional mechanical demands. Parameters such as pore size, porosity, and trabecular thickness are precisely modulated by adjusting seed point distribution and density. To ensure precise microstructural adjustments, geometric characteristics of natural trabeculae—including morphology, spatial distribution, anisotropy, and spatial randomness—are meticulously replicated. As a result, an anisotropic porous scaffold with a 600 µm pore size and 70% porosity was fabricated successfully. Compared to conventional porous structures (e.g., cubic, cylindrical, and triply periodic minimal surfaces), the Voronoi‐based structure demonstrates enhanced complexity and morphological diversity, more closely mimicking natural trabeculae. Conventional porous structures are typically isotropic, limiting their ability to meet the directional mechanical demands of bone tissue.^[^ ^18^ ^]^ The anisotropic properties of the Voronoi structure, optimized through image data integration, support scaffold designs that satisfy direction‐specific mechanical requirements. Consequently, this integrated approach enhances the scaffold's mechanical adaptability and biological compatibility, rendering it suitable for diverse loading conditions.

We successfully employed 3D printing technology to fabricate a porous Ti‐6Al‐4V scaffold with a biomimetic trabecular structure. Finite element analysis and mechanical testing reveal the scaffold's robust mechanical properties, particularly its high strength and low elastic modulus. Importantly, the scaffold exhibits an elastic modulus of 3.2 GPa, markedly lower than the conventional Ti‐6Al‐4V modulus of ≈110 GPa. A high elastic modulus in traditional implants commonly induces stress shielding, thereby hindering bone tissue regeneration.^[^ ^19^ ^]^ By contrast, the scaffold's elastic modulus approximates that of natural cancellous bone, thereby enhancing biomechanical compatibility. This mechanical match minimizes stress shielding, fosters a conducive microenvironment, and optimizes stress transfer to new bone tissue, thereby mitigating issues like bone resorption and implant loosening commonly seen in standard Ti‐6Al‐4V implants. These findings are consistent with those of Wieding et al.,^[^ ^20^ ^]^ who found that optimizing porous Ti‐6Al‐4V scaffold geometry effectively reduces the elastic modulus, aligning with bone mechanics and enhancing osseointegration. Additionally, CFD analysis demonstrated favorable permeability (11.52 × 10^−8^ mm^2^) and uniform fluid distribution across the porous scaffold. The scaffold's porous structure not only promotes cell adhesion, proliferation, and differentiation but also facilitates vascular and nutrient infiltration. This is supported by enhanced cell viability, proliferation, and angiogenic marker expression in vitro. These features are crucial for supporting cellular growth, neovascularization, and tissue regeneration, aligning with the principles of effective bone tissue engineering. These attributes encourage neovascularization and tissue regeneration, upholding the principle of “structure‐function unity” central to bone tissue engineering. Uniform fluid shear and pressure distribution simulate the natural bone marrow environment, further promoting bone regeneration. This observation aligns with Foroughi et al.,^[^ ^21^ ^]^ who emphasized the essential role of fluid mechanics in porous scaffolds for tissue engineering.

Building upon the optimized structural and mechanical framework, pDA was employed as a surface modifier to enhance the scaffold's synergy with bioactive compounds. pDA exhibits adhesion properties akin to mussel foot proteins, allowing a stable coating to form on the scaffold surface. This coating supplies a rich density of active functional groups, which facilitates the binding of bioactive molecules.^[^ ^22^ ^]^ SEM and EDS analyses confirmed both effective pDA modification and the successful integration of PGHExo microspheres on the BTPS surface. The addition of pDA significantly enhances BTPS biocompatibility, creating an ideal interface for anchoring exosome‐loaded double‐network hydrogel microspheres. Cell viability and proliferation assays indicated markedly higher cell survival and growth rates in the BTPS&pDA group compared to the unmodified BTPS group. Consistent with findings by Wang et al.,^[^ ^23^ ^]^ these results further validate that pDA coatings enhance the biofunctionalization of scaffold materials.

The bioactive properties of the porous structure in biomimetic trabecular bone are crucial for facilitating osteogenesis and angiogenesis.^[^ ^24^ ^]^ Importantly, the development and application of PGHExo, a dual‐network hydrogel microsphere engineered for controlled release under hypoxic conditions in HUVECs, marks a notable innovation in this study. HUVEC‐derived exosomes were successfully isolated and analyzed under both normoxic and hypoxic conditions using a multi‐step, high‐speed centrifugation protocol. Moreover, the results revealed that hypoxia markedly increased the protein content, particle concentration, and cellular uptake of exosomes, thus enhancing their angiogenic potential. Notably, this pro‐angiogenic effect was dose‐dependent, indicating that higher exosome concentrations could amplify angiogenic responses. However, at higher concentrations (200 µg mL^−1^), a saturation effect emerged, constraining further increases in angiogenic activity. Consistent with prior research, these findings illustrate that hypoxia upregulates VEGF expression and augments exosome release, reinforcing hypoxia's pivotal role in angiogenesis.^[^ ^25^ ^]^ Utilizing microfluidic technology, we successfully fabricated dual‐network hydrogel microspheres embedding Hypo‐Exo within a PEGDA/GelMA matrix, enabling controlled, sustained exosome release. Characterization of PGHExo microspheres revealed a uniform particle size, high encapsulation efficiency, and controlled release profile, validating the efficacy of microfluidic technology. Compared to conventional exosome delivery methods, the PGHExo system extended exosome release significantly, sustaining release for up to 18 days. This extended‐release sharply contrasts with the brief 5–7‐day release profiles associated with direct injection or electrospinning techniques.^[^ ^26^ ^]^ This controlled‐release system ensured prolonged osteogenic and angiogenic stimulation in both in vitro and in vivo environments.

Exosomes are essential mediators of intercellular communication, transporting various bioactive molecules—such as microRNAs, proteins, and lipids—that collectively regulate cellular processes.^[^ ^27^ ^]^ In our in vitro studies, MC3T3‐E1 osteoblasts exposed to BTPS&pDA@PGHExo exhibited the highest proliferation, ALP activity, mineralized nodule formation, and upregulation of osteogenesis‐related genes compared with all other groups. These effects likely arise from the activation of osteogenic signaling pathways by specific exosomal microRNAs and proteins. Moreover, qRT‐PCR revealed significantly elevated mRNA levels of angiogenesis‐related genes (e.g., VEGF, CD31, PDGFB, FGF2) in the BTPS&pDA@PGHExo group, suggesting that exosomal pro‐angiogenic factors such as VEGF and FGF2 enhance endothelial cell function and augment angiogenic capacity. Given that angiogenesis is crucial for bone regeneration—providing essential nutrients and oxygen—these findings support the notion that exosomes effectively coordinate osteogenic and angiogenic processes. Consistent with this, mRNA‐seq analyses demonstrated elevated expression of osteogenesis‐related genes (e.g., ALPL, COL18A1, SAMD6, RUNX2OS1) and angiogenesis‐related genes (e.g., VEGFB, PDGFA, ANGPT2) associated with pathways including MAPK, mTOR, HIF‐1, and VEGF. These results were further validated by the qRT‐PCR and ELISA experiments, which confirmed that the observed gene expression changes were reflected at the protein level. Collectively, these data indicate that BTPS&pDA@PGHExo enhances osteogenic differentiation by orchestrating multiple signaling pathways. Additionally, Exosomes may also promote tissue repair by modulating mitochondrial function, inflammation, and macrophage polarization states.^[^ ^28^ ^]^

Crucially, the hypoxia‐induced endothelial exosomes embedded within our dual‐network hydrogel microspheres contain a rich cargo of bioactive molecules, including specific microRNAs (e.g., miR‐210, miR‐21) and proteins (e.g., VEGF, FGF2), known to drive osteogenic and angiogenic responses.^[^ ^29^ ^]^ Under hypoxic conditions, stabilized HIF‐1α in endothelial cells enhances the packaging of these regulatory miRNAs and proteins into exosomes, which are then internalized by osteoprogenitor and endothelial progenitor cells. These exosomal miRNAs modulate key pathways such as HIF‐1, VEGF, and mTOR, facilitating the proliferation, migration, and differentiation of cells essential for bone and vascular formation.^[^ ^30^ ^]^ For instance, miR‐210 upregulates HIF‐1 target genes, increasing VEGF secretion and promoting neovascularization while simultaneously influencing osteoblast lineage commitment and matrix mineralization.^[^ ^31^ ^]^ Similarly, the protein cargo of these exosomes can directly activate pro‐angiogenic signaling cascades, enhancing vascular ingrowth and tissue perfusion.^[^ ^32^ ^]^ By integrating these hypoxia‐induced exosomes into PEGDA/GelMA dual‐network hydrogel microspheres, our approach ensures their spatially and temporally controlled release, maintaining a sustained, localized regulatory influence on the host microenvironment. These findings align with prior evidence that exosomes modulate cellular metabolism, inflammatory responses, and macrophage polarization—key elements of the regenerative cascade.^[^ ^33^ ^]^ This precisely engineered, molecularly coordinated strategy underpins the enhanced osteogenesis and angiogenesis observed in our experiments, broadening our understanding of how biomaterials can harness endogenous signaling networks to promote functional tissue regeneration. In a preclinical in vivo rabbit femoral defect model, the BTPS&pDA@PGHExo scaffold exhibited notable efficacy in promoting bone repair. Micro‐CT and histological analyses further revealed that, at 4 and 12 weeks post‐surgery, the BTPS&pDA@PGHExo group displayed significantly greater bone volume and density than control groups. Additionally, the defect region in this group exhibited extensive new trabecular bone formation and an organized vascular network. Taken together, these findings underscore the pivotal role of the dual‐network hydrogel sustained‐release microsphere loaded with hypoxia‐induced HUVEC exosomes in facilitating vascularized bone regeneration. These results align with previous findings by Hu et al.,^[^ ^34^ ^]^ which underscore the critical role of exosomes in bone tissue regeneration.

Hence, this study's scaffold system offers several critical advantages over conventional Ti‐6Al‐4V scaffolds. Specifically, the biomimetic design, enabled by advanced 3D printing, allows the scaffold's mechanical properties to closely resemble natural bone, effectively minimizing the mechanical mismatch with host tissue. Moreover, the incorporation of sustained‐release dual‐network hydrogels, driven by hypoxia‐induced HUVEC‐derived exosomes, markedly enhances scaffold bioactivity, promoting vascularized bone formation. Finally, the versatility of 3D printing technology allows precise scaffold customization, addressing the specific anatomical and functional needs of individual patients.

While encouraging, these promising results come with certain inherent limitations. The molecular pathways by which exosomes facilitate bone regeneration are not yet fully delineated, underscoring the need for additional studies to achieve a comprehensive understanding. Furthermore, the limited duration of the animal experiments constrains our understanding of the scaffold's long‐term efficacy and stability. Future investigations should focus on elucidating the specific regulatory mechanisms of exosomes at cellular and molecular levels. Additionally, larger‐scale animal studies and preclinical evaluations are planned to thoroughly assess the feasibility and safety of this therapeutic strategy.

## 4 Conclusion

Herein, we establish a dual biomimetic paradigm that integrates a structurally inspired 3D‐printed Ti‐6Al‐4V trabecular scaffold with an HExo biochemical cue to facilitate vascularized bone regeneration. By meticulously adjusting both the trabecular anatomical morphology and mechanical properties of the scaffold to closely mimic native cancellous bone, and by enabling prolonged exosome release through a PEGDA/GelMA dual‐network hydrogel, our approach mitigates stress shielding, enhances cellular infiltration, and sustains osteogenic and angiogenic signaling over clinically relevant timescales. Beyond yielding improved bone volume, mineralization, and neovascularization in vivo, this platform addresses critical hurdles in treating large bone defects and complex skeletal injuries, and may be extended to other tissues requiring concurrent mechanical and vascular integration. Future investigations will delve deeper into the molecular underpinnings of exosome‐mediated regeneration, harness patient‐specific anatomies for personalized scaffold design, and ensure long‐term safety and functional stability. Collectively, our findings mark a pivotal step toward next‐generation, bioinspired implants that seamlessly integrate into host tissues, bridging the gap between engineered constructs and the natural complexity of human bone.

## 5 Experimental Section

### Design of the BTPS

To develop a biomimetic trabecular porous structure reflecting human anatomical characteristics, this study first employs CT scanning to obtain high‐resolution images of trabecular bone, thereby enabling the extraction of microstructural details and subsequent 3D modeling. Subsequently, Rhino software (McNeal, Seattle, W A, USA) and its Grasshopper (v.0.9.0076) plug‐in were employed to design a parameterized porous structure based on the Voronoi algorithm. Real‐time adjustments to the algorithm parameters ensure that the porous structure closely replicates the natural anatomical configuration and spatial organization of the femoral trabecular bone. With reference to the design methodology of the previous study,^[^ ^15^ ^]^ finite element and fluid mechanics analyses optimized a pore size of 600 µm and a porosity of 70% in the anisotropic biomimetic trabecular porous structure. This design not only replicates the intricate geometry of natural bone trabeculae but also offers substantial mechanical and biological benefits, including enhanced mechanical adaptability and a supportive biological microenvironment. The detailed optimization process of the biomimetic trabecular porous structure design is provided in Figure S1 (Supporting Information).

### Preparation and Characterization of BTPS via 3D Printing

The BTPS was fabricated in the United Kingdom using a Renishaw metal 3D printer with Ti‐6Al‐4V ELI‐0406 powder. To prevent oxidation, the powder was protected by an argon gas atmosphere throughout the printing process. SLM technology was employed under precise conditions, including a 70 µm laser spot diameter, a 170 °C substrate temperature, 30 µm layer thickness, and a 67° rotation per layer. Additionally, the laser scanning speed was adjusted based on dot spacing, exposure time, and drill delay to ensure optimal printing quality. Following the printing process, the excess powder was removed, and post‐processing produced a BTPS with dimensions of 10 mm in both height and diameter. For the cell culture, an additional BTPS sample was prepared with a height of 2 mm and a diameter of 10 mm. The surface morphology of the scaffold was characterized by SEM, while EDS was used to map the elemental distribution. The internal structure accuracy was further verified by high‐resolution industrial CT scanning (RX Solutions, Wuxi Raider Detection Technology Co., Ltd., Wuxi, China). Quantitative analysis of porosity, pore size, and trabecular thickness was conducted using Avizo and VG Studio Max (Wuxi Rui de Inspection Technology Co., Ltd., Wuxi, China) software.

### Finite Element Analysis of BTPS

The primary aim of this experiment was to evaluate and refine the mechanical properties of the BTPS. First, a comprehensive 3D solid model of BTPS was constructed in 3‐Matic software (Materialise NV, Belgium, Version 11.0), followed by generating a high‐quality surface and volume mesh. Subsequently, Ti‐6Al‐4V material properties (elastic modulus 110 GPa, Poisson's ratio 0.3)^[^ ^35^ ^]^ were assigned in Mimics (Materialise NV, Belgium, Version 19.0), and the model was exported in.cbd format to ANSYS Workbench 18.0 (ANSYS, Inc., USA) to establish the finite element model. To accurately simulate physiological loading conditions, fixed constraints were applied at the lower end of the BTPS model, while an axial compression load of 500 N was introduced at the upper end. Finally, the finite element analysis sought to analyze the stress distribution and displacement response of the BTPS under load, thereby identifying areas for optimization.

### CFD of BTPS

CFD simulations were performed to investigate the fluid dynamics characteristics of the BTPS. Simulations were conducted using Ansys software, employing the Navier–Stokes Equation (1) to model incompressible fluid flow. Water was selected as the working fluid, characterized by a density of 1000 kg m^−^^3^ and a viscosity of 1.002 × 10⁻^3^ Pa·s.^[^ ^36^ ^]^ Key fluid dynamic parameters, including shear force, pressure distribution, and flow velocity, were analyzed throughout the BTPS. The Reynolds number (Re), calculated using Equation (2), was employed to distinguish between laminar and turbulent flow states.^[^ ^37^ ^]^ Permeability (K), as calculated by Equation (3), was employed to assess mass transfer capabilities across distinct porous configurations. To minimize boundary effects, an additional fluid domain was established above the BTPS model, with an inlet flow rate set at 1 mm s^−1^ and an outlet pressure maintained at zero. A no‐slip boundary condition was imposed on the BTPS walls to accurately model realistic flow behavior. The pressure difference (ΔP) was computed following Equation (4). This simulation approach accurately captures the fluid dynamics environment within the BTPS, thereby facilitating design optimization.

(1) $$\rho \left(\frac{\partial u}{\partial t}+u\cdot \nabla u\right)=-\nabla p+\mu \nabla^{2}u+F\;\nabla \cdot u=0$$

where u is the velocity of the fluid (m s^−1^); ρ is the density of the fluid (kg m^−3^); t is the time (s); *p* is the pressure (Pa); µ is the dynamic viscosity coefficient of the fluid (Pa s); ∇ is the operator; F is the acting force (N).

(2) $$Re=\frac{\rho vL}{\mu }$$

(3) $$K=\frac{v\cdot \mu \cdot L}{\Delta P}$$

(4) $$\Delta P=P_{\mathrm{inlet}}-P_{\mathrm{outlet}}$$

where *Re* is the Reynolds number; 𝑣 is the flow velocity(m s^−1^); L is the characteristic length (mm); K is the permeability coefficient (mm^2^); ΔP is the pressure difference (MPa); *P*_inlet_ is the pressure at the inlet (MPa); *P*_outlet_ is the pressure at the outlet (MPa).

### Mechanical Analysis of the BTPS

The mechanical properties of the BTPS were rigorously assessed through a static axial compression test conducted on a universal testing machine (WDW‐100Y, Changzhou Geasure Medical Apparatus and Instruments Co., Ltd). During testing, each sample was securely positioned in a metallic holder. The pressure was then applied through a hollow push rod connected by a universal joint, with a loading rate set at 1 mm min^−1^, until the sample exhibited either deformation or fracture, indicating structural failure. For each experiment, a minimum of three samples were tested, with load‐displacement and stress‐strain curves recorded to derive the yield load, elastic modulus, and comprehensive mechanical profile of the BTPS.

### Extraction and Characterization of HUVECs‐Derived Exosomes—Extraction of Exosomes

HUVECs were initially cultured in Dulbecco's Modified Eagle's Medium (DMEM, Gibco) supplemented with 10% fetal bovine serum under both normoxic and hypoxic conditions. Upon reaching ≈80% confluence, the medium was replaced with exosome‐free DMEM and the culture was continued for another 48 h. Following incubation, exosome‐rich supernatants were separately collected from both normoxic and hypoxic cultures. Subsequently, exosomes were isolated through a multi‐step centrifugation process as previously established.^[^ ^38^ ^]^ Specifically, centrifugation steps at 300 g, 2000 g, 10 000 g, and 100 000 g were performed to successively eliminate live/dead cells, debris, and larger particles. Then, exosomes were resuspended in phosphate‐buffered saline(PBS, Gibco). Finally, exosomes derived from normoxic and hypoxic HUVEC cultures were designated as normoxia exosomes (Exo) and hypoxia exosomes (Hypo‐Exo), respectively.

### Extraction and Characterization of HUVECs‐Derived Exosomes—BCA Protein Quantification

Each exosome sample (20 µL) was combined with BCA (Thermo Scientific) working solution and incubated at 37 °C for 30 min. Following incubation, the absorbance at 562 nm was recorded using a microplate reader. Using a standard curve, the protein concentration was calculated, and all samples were adjusted to equivalent protein concentrations for consistent downstream analyses.

### Extraction and Characterization of HUVECs‐Derived Exosomes—Exosome Marker Identification via Western Blot

Adhering to the Minimal Information for Studies of Extracellular Vesicles (MISEV2018) standards, exosome‐specific markers were identified by Western blotting. Each 100 µL exosome sample was combined with 20 µL loading buffer, heated to 95 °C for 5 min, and subsequently subjected to SDS‐PAGE. After electrophoresis, proteins were transferred to a PVDF membrane, which was blocked in 5% PBS‐based skim milk for 1 h at room temperature. The membrane was then incubated with primary antibodies (anti‐CD81(ab109201, Abcam), anti‐CD9(ab223052, Abcam), anti‐ALIX(ab117600, Abcam), anti‐β‐actin(ab8227, Abcam)) diluted 1:10 000 at 4 °C overnight, followed by a 1 h incubation with a 1:5000 horseradish peroxidase‐conjugated secondary antibody (ab205718, Abcam) at room temperature. To visualize, an ECL reagent was applied, and chemiluminescent signals were imaged after a 3 min exposure.

### Extraction and Characterization of HUVECs‐Derived Exosomes—NTA

A 1 mL volume of each diluted exosome sample was injected into the Nanosight nanoparticle analyzer, and the temperature probe was positioned accordingly. Once focused, the particle size distribution and concentration were determined at a wavelength of 405 nm, with data subsequently recorded for analysis.

### Extraction and Characterization of HUVECs‐Derived Exosomes—TEM Characterization

To examine the morphology of exosomes, TEM imaging was conducted on a Hitachi H‐7650 microscope set at 80 kV.

### Uptake of Exos by HUVECs—Exosome Labeling

For fluorescence labeling, Exos and Hypo‐Exos were treated with PKH26 (Sigma–Aldrich). Specifically, 6 µL of PKH26 dye was added to 1 mL of diluted exosome solution, mixed gently, and incubated at room temperature for 5 min. Next, 2 mL of 10% BSA quenching solution was added, and the total volume was brought to 8.5 mL with serum‐free medium. The mixture was then transferred to an ultracentrifuge tube and subjected to centrifugation at 190 000 *g* for 2 h at 4 °C. Afterward, PKH26‐labeled exosomes were carefully resuspended in Dulbecco's phosphate‐buffered saline (DPBS) under dark conditions, quantified using a BCA kit, and stored at −80 °C.

### Uptake of Exos by HUVECs—Exosome Uptake

To evaluate exosome uptake by HUVECs, 200 µg mL^−1^ of PKH26‐labeled exosomes were added to the HUVEC medium and incubated separately for 24 h. After each incubation period, cells were washed with DPBS, fixed in 4% paraformaldehyde (PFA) for immunostaining, and subsequently stained with 4′,6‐diamidino‐2‐phenylindole (DAPI, Invitrogen) for nuclear visualization. For each group, three representative samples were imaged using an LSM880 confocal laser scanning microscope (Zeiss), and ImageJ software (National Institutes of Health, USA) was employed to calculate the relative fluorescence intensity.

### Hypo‐Exos Concentration Screening

To determine the optimal Hypo‐Exos concentration for angiogenesis enhancement, angiogenesis experiments were conducted. Hypo‐Exos were diluted to final concentrations of 50, 100, 150, and 200 µg mL^−1^. HUVECs were seeded into 96‐well plates pre‐coated with Matrigel and treated with Hypo‐Exos at each specified concentration. Following incubation at 37 °C for 6–8 h, the formation of vascular‐like structures was observed and photographed. Quantitative analysis of angiogenesis was conducted by calculating the total mesh area in images using ImageJ software, enabling comparison across Hypo‐Exos concentrations.

### Preparation of PGHExo Microfluidic Devices

To prepare PGHExo double‐network hydrogel sustained‐release microspheres, microfluidic chip technology was employed. Initially, the primary channel of the microfluidic device was loaded with a prepolymer solution composed of 1% PEGDA (Sigma–Aldrich), 1% GelMA (Sigma–Aldrich), 1‐3 mg mL^−1^ Hypo‐Exos, and 0.5% w/v Lithium phenyl‐2,4,6‐trimethylbenzoylphosphinate (LAP, Sigma–Aldrich). Concurrently, mineral oil (containing 10% span 80) functioned as the shear phase. The two‐phase flow was carefully regulated at respective flow rates of 200 and 1000 µL h^−1^ to ensure uniform microsphere formation. Subsequently, microspheres underwent crosslinking and solidification within the microfluidic device via 365 nm UV exposure for 15 s. Following solidification, microspheres were rinsed with deionized water and transferred to a 1.5 mL Eppendorf tube. Subsequently, centrifugation at 5000 rpm for 15 min was performed on the microspheres, repeating this step three times. Finally, light microscopy assessment was conducted, after which the microspheres were resuspended in deionized water and stored at 4 °C.

### Microstructure and Rheological Properties of PGHExo Microspheres—SEM

The PGHExo microspheres were freeze‐dried and subsequently coated to enhance electrical conductivity. Next, the surface morphology of the microspheres was examined under magnifications of 200x and 2000x. Microsphere particle sizes were analyzed using ImageJ software, generating a detailed particle size distribution map.

### Microstructure and Rheological Properties of PGHExo Microspheres—Rheological Properties

A rheometer (TA Instruments) was employed to quantify the storage modulus (G′) and loss modulus (G″), thereby characterizing the PEGDA/ GelMA gel's mechanical properties. Testing frequencies were varied from 0.1 to 10 rad s^−1^ while maintaining a constant temperature of 37 °C. Under these controlled conditions, both GelMA (1%) and PEGDA/ GelMA (1%) samples were analyzed to ascertain their frequency‐dependent rheological properties.

### Release Characteristics of PGHExo Microspheres—BCA Analysis

The release profile of Hypo‐Exos from PGHExo microspheres was evaluated in a simulated body fluid (SBF, Leagene, CZ0403) solution. Each microsphere group was immersed in 1 mL of degradation solution and then continuously agitated at 37 °C. Protein concentration was measured every three days up to day 18 by mixing 20 µL of solution with an equivalent volume of degradation solution. At each measurement interval, 20 µL of fresh degradation solution was added. BCA assays were conducted on days 0, 3, 6, 9, 12, 15, and 18. To maintain protein concentration consistency after each measurement, an equal volume of BSA solution was added to the SBF. Additionally, exosome release concentrations were quantified. Protein concentrations were determined using a BCA kit, with each group measured in triplicate. The release rate was calculated as follows (Equation 5):

(5) $$\mathrm{Release}\;\mathrm{rate}\;\left(\%\right)=\left(C_{t}\;-\;C_{0}\right)\;/\;C_{l}\;\times \;100$$

where C_0_ denotes the protein concentration of PG microspheres without exosomes, C_t_ denotes the measured concentration for each group, and C_l_ represents the initial exosome loading concentration.

Additionally, excluding day 0, the protein concentration measured at the previous time point was defined as C_t‐1_. The three‐day sustained release concentration was then calculated using the following formula (Equation 6):

(6) $$\mathrm{Protein}\;\;\mathrm{concentration}\;\left(\mathrm{mg}/\mathrm{mL}\right)=C_{t}\;-\;C_{t-1}$$

### Release Characteristics of PGHExo Microspheres—Fluorescence Analysis

To enable tracking of Hypo‐Exos release from the microspheres, PKH26‐labeled Hypo‐Exos (2 mg mL^−1^) were introduced into the aqueous phase. Consistent with this approach, PGHExo microspheres were prepared using the same protocol. Subsequently, a specified quantity of PGHExo microspheres was introduced into 2 mL of SBF, allowing for individual monitoring of each microsphere. Fluorescence signals were then recorded using an LSM880 confocal laser scanning microscope on days 0, 3, 6, 9, 12, 15, and 18. Images were analyzed with ZEN software to quantify both relative fluorescence intensity and microsphere volume.

### Modification of BTPS with Embedded PGHExo Microspheres and Characterization

A pDA (Sigma–Aldrich) solution, prepared at pH 8.5 with a concentration of 2 mg mL^−1^ in 300 mL of 10 mm Tris‐HCl (Sigma–Aldrich), was synthesized. Subsequently, the 3D‐printed BTPS was immersed in the pDA solution and agitated overnight in a light‐protected environment. Afterward, the BTPS was removed, rinsed thrice with deionized water, and dried at 37 °C for subsequent application. BTPS was then positioned in a well plate, with 1 mL of PGHExo microspheres resuspended in 500 µL of PBS. The BTPS surface was sonicated and maintained in a sterile environment for 24 h, enabling the PGHExo microspheres to embed onto the pDA film, thus forming the BTPS&pDA@PGHExo composite. The morphology and elemental composition of BTPS&pDA@PGHExo were comprehensively characterized using SEM to assess its structural and compositional properties.

### Cell Culture

This study employed the MC3T3‐E1 mouse cranial osteoblast cell line and HUVECsq, both sourced from the Chinese Academy of Sciences Cell Bank. For optimal growth, the culture medium for each cell type was supplemented with 10% fetal bovine serum, 100 µg mL^−1^ streptomycin, and 100 U mL^−1^ penicillin. All procedures were conducted aseptically within a biosafety cabinet to maintain sterile conditions. Additionally, all reagents, including DPBS, culture medium, and 0.2% trypsin, were preheated and prepared in advance. Following this, cells in the logarithmic growth phase were washed twice with DPBS, digested using 0.2% trypsin and neutralized by adding a complete medium. The resulting cell suspension was then transferred to a 15 mL centrifuge tube, centrifuged at 800 rpm for 5 min, after which the supernatant was discarded. Subsequently, the cells were resuspended, counted, and adjusted to the target concentration. To prepare for cell seeding, pre‐sterilized scaffolds were placed in a 24‐well culture plate and incubated overnight at 37 °C in a 5% CO₂ atmosphere. Finally, MC3T3‐E1 cells were seeded onto the scaffolds at a density of 5 × 10⁴ cells per scaffold, setting the stage for subsequent experimental procedures.

### Cell Viability Assay

To evaluate cell viability, MC3T3‐E1 cells were assessed using a Live/Dead cell viability kit (Invitrogen, USA), allowing precise determination of live‐to‐dead cell ratios across groups. Cells were seeded onto BTPS, BTPS&pDA, BTPS&pDA@PG, and BTPS&pDA@PGHExo scaffolds and cultured for 24 h. The scaffolds were then carefully rinsed with DPBS two or three times to remove any residual medium. The staining solution, containing Calcein AM and Ethidium homodimer‐1 diluted in DPBS at a 1:1 to 1:1000 ratio, was prepared in darkness to ensure optimal staining efficacy. A total of 500 µL staining solution was added to each well and incubated at room temperature for 30 min, after which the solution was discarded and washed three times with DPBS. The samples were subsequently visualized under a fluorescence microscope within a live cell workstation, capturing and documenting the distribution of live (green fluorescence) and dead (red fluorescence) cells. Background noise was delineated using a dashed line to identify noise areas, thereby minimizing the impact of background interference. Statistical regions were manually defined, and cell viability was subsequently quantified using ImageJ.

### Cell Proliferation Assay

The Alamar Blue assay was employed to quantify MC3T3‐E1 cell proliferation on each scaffold type. MC3T3‐E1 cells were seeded at a density of 5 × 10⁴ cells per scaffold across BTPS, BTPS&pDA, BTPS&pDA@PG, and BTPS&pDA@PGHExo scaffolds, with proliferation measured at 1, 3, and 5 days. Subsequently, the samples were washed with DPBS and transferred to new 24‐well plates. At each designated time point, a 1:10 mixture of Alamar Blue solution (Invitrogen, USA) and complete medium was introduced to each well. The samples were incubated at 37 °C in darkness for 4 h. Following incubation, the supernatant was carefully collected and transferred to a 96‐well plate. Finally, cell proliferation was quantified using a fluorescence detection system (Cytation5, BioTek, USA) with excitation and emission wavelengths of 530 and 590 nm, respectively.

### Cell Osteogenic ALPAssay

The influence of different scaffolds on the osteogenic differentiation of MC3T3‐E1 cells was systematically evaluated. After seeding cells onto each scaffold and incubating them for 24 h, cultures were then maintained in the osteogenic induction medium (high‐glucose DMEM with 10% FBS, 100 µg mL^−1^ streptomycin, 100 IU mL^−1^ penicillin, 0.1 µM dexamethasone, 10 mm β‐glycerophosphate, and 50 µg mL^−1^ sodium ascorbate) for 7 days. The medium was refreshed every 2 to 3 days to sustain optimal conditions for differentiation. On the seventh day, supernatants from the cell lysates were collected and analyzed for ALP activity using an ALP detection kit (Beyotime, China), thereby quantifying the degree of osteogenic differentiation.

### Alizarin Red S Mineralization Assay

To assess the mineralization capacity of osteoblasts, cells were cultured under a standardized osteogenic induction protocol for 21 days. Following the incubation, the medium was discarded, and cells were washed 2–3 times with DPBS before fixation in 4% paraformaldehyde. Subsequently, cells were stained at room temperature with a 1% Alizarin Red S solution (Sigma–Aldrich). After staining, the solution was discarded, and cells were washed thoroughly with DPBS until no visible dye residue was observed. The scaffolds were then dried at 37 °C to document the morphology of mineralized nodules. Next, the scaffolds were transferred to a 6‐well plate, and a 10% hexadecylpyridinium chloride solution (Sigma–Aldrich) was added to elute the dye under shaking conditions at 37 °C and 100 rpm. Finally, absorbance at 620 nm was recorded using a microplate reader (Thermo Fisher, USA) to quantify the formation and distribution of calcium‐mineralized nodules.

### qRT‐PCR Analysis—Osteogenic Gene Expression

RT‐PCR was employed to quantify osteogenic gene expression. MC3T3‐E1 cells were seeded on each scaffold group and cultured for 7 days. Total RNA was extracted using Trizol reagent (Beyotime, China) according to the manufacturer's instructions, and its concentration and purity were assessed with a NanoDrop spectrophotometer (Thermo Fisher Scientific, USA). Reverse transcription of RNA into complementary DNA (cDNA) was performed using a commercially available kit (Beyotime, China). Quantitative PCR was conducted using a SYBR Green PCR kit (Thermo Fisher Scientific, USA) on an RT‐PCR system (Beyotime, China). Primers were designed to target osteogenic markers Runx2, ALP, OCN, and COL1A1, with GAPDH serving as the internal reference gene. The primer sequences are listed in Table S1 (Supporting Information). The thermal cycling conditions were set as follows: initial denaturation at 95 °C for 5 min, followed by 40 cycles of 95 °C for 30 s, 60 °C for 30 s, and 72 °C for 30 s. Relative gene expression levels were calculated using the 2^(‐ΔΔCt)^ method and statistically analyzed to determine the effects of scaffolds on osteoblast‐related gene expression.

### qRT‐PCR Analysis—Angiogenic Gene Expression

Angiogenic gene expression was assessed using qRT‐PCR to evaluate the effects of the scaffolds on vascularization. HUVECs were seeded onto each scaffold group and cultured for 3 days. The expression levels of VEGF, CD31, PDGFB, and FGF2 were quantified by RT‐PCR, with the primer sequences provided in Table S1 (Supporting Information). The experimental procedures and data analysis were performed following the protocol described for osteogenic gene expression, using GAPDH as the internal reference gene.

### mRNA‐seq Analyses

The MC3T3‐E1 cell line was utilized to investigate the underlying mechanisms. Cells were cultured on scaffolds under standard conditions until the predefined incubation period was completed. Total RNA was extracted using a TRIzol reagent following the manufacturer's protocol. The quality and purity of RNA were assessed with a NanoDrop spectrophotometer and validated via agarose gel electrophoresis to ensure suitability for library preparation. High‐quality RNA was reverse‐transcribed into cDNA, and cDNA libraries were prepared for high‐throughput sequencing on the Illumina platform (Zhejiang, China). The sequencing data underwent rigorous quality control, alignment to the reference genome, gene expression quantification, and identification of differentially expressed genes. GO and KEGG enrichment analyses were performed on key genes to uncover essential osteogenesis‐related pathways, offering insights into the scaffold's role in regulating bone formation. Furthermore, the expression levels of bone‐related genes (ALPL, COL18A1, SAMD6, RUNX2OS1) and angiogenic genes (VEGFB, PDGFA, ANGPT2) were analyzed following the experimental protocol described under the subhead “qRT‐PCR Analysis” to validate the differentially expressed genes (DEGs) identified in the mRNA‐seq analysis. The primer sequences are provided in Table S1 (Supporting Information).

### ELISA

The concentrations of osteogenic markers (RUNX2, OCN) and angiogenic markers (PDGF, VEGF) in the cell supernatant were quantified using species‐specific ELISA kits (ELK Biotechnology) according to the manufacturer's instructions. Briefly, centrifuged serum samples (3000 × g, 15 min) from each treatment group were incubated in antibody‐precoated wells, followed by incubation with the corresponding detection antibodies. The absorbance was measured at 450 nm using a microplate reader, and concentrations were determined based on standard curves. To ensure data accuracy and reproducibility, all measurements were performed in technical triplicates.

### Animals and Surgical Procedures

All animal care and experimental procedures were performed in strict accordance with institutional guidelines, as well as national laws and regulations. The animal experimental protocol was reviewed and approved by the Animal Ethics Committee of Southern Medical University (SMUL2023091), and all animal experiments complied fully with the committee's ethical requirements. Healthy adult New Zealand white rabbits (2.5–3.0 kg) were acclimated for one week and then randomly assigned to one of four groups (*n* = 6 per group, totaling 24): BTPS, BTPS&pDA, BTPS&pDA@PG, and BTPS&pDA@PGHExo. Prior to the procedure, general anesthesia was induced with 3% sodium pentobarbital at a dose of 1 mL kg^−1^. Once anesthesia was confirmed, each rabbit was secured on the operating table, and the hindlimbs were shaved and disinfected according to standard protocols. A longitudinal incision above the knee joint was made to expose the distal femur, ensuring aseptic techniques throughout. A bone defect model with a diameter and depth of 10 mm was drilled into the distal femur, carefully avoiding adjacent soft tissues and vital structures. Following thorough saline irrigation of the defect, the corresponding scaffold was implanted based on the assigned grouping. The muscles, subcutaneous tissue, and skin were sutured in sequential layers. To prevent infection, the wound was irrigated with saline and an antibiotic solution, supplemented with routine postoperative care, including antibiotic injections and wound management. Postoperative monitoring of behavior and wound healing was conducted to ensure no signs of infection or complications.

### Micro‐CTAnalysis

Micro‐CT imaging was performed on the implantation sites at 4 and 12 weeks post‐surgery using a Bruker 1276 Micro‐CT scanner to evaluate the repair progress of femoral bone defects in rabbits. Following euthanasia, femoral bone specimens containing implants were carefully extracted, rinsed thoroughly with saline, and fixed in 10% neutral formalin for 24 h to preserve tissue structure. The specimens were subsequently rinsed to eliminate any residual fixative solution before undergoing Micro‐CT scanning. Scanning parameters were set at 70 kV for voltage, 200 µA for current, with a resolution of 9 µm and an exposure time of 200 ms, encompassing the bone defect and adjacent normal bone tissue. The acquired scan data were processed using 3D reconstruction software to obtain high‐resolution images of the bone tissue. Subsequently, image analysis software was applied to quantify new bone volume and density, providing a comprehensive assessment of bone repair efficacy.

### Histopathological Analysis

To assess histopathological features of bone defect repair in the rabbit distal femur, samples from the implant site were collected at 4 and 12 weeks post‐surgery. Samples were rinsed in saline and immediately fixed in 10% neutral formalin for 48 h to ensure tissue structure preservation. Fixed samples were subsequently dehydrated in 70%, 80%, 90%, 95%, and finally 100% ethanol (each concentration applied twice) and embedded in polymethyl methacrylate (PMMA, Cool‐Set‐A, OLYMPUS, Chengdu). Tissue sections, 10–20 µm thick, were prepared using a diamond microtome (SAT‐001, Olinkem, Chengdu) and subsequently stained with methylene blue and basic fuchsin (Sigma) to examine histological structures and cellular morphology. Stained sections were then observed and imaged using an optical microscope, with a primary focus on new bone and blood vessel formation. Image analysis, conducted via ImageJ software, quantitatively assessed new bone and blood vessel distribution, quantity, and morphology to comprehensively characterize bone repair outcomes.

### Statistical Analysis

Experimental data were reported as the Mean ± SD, with each group consisting of at least three independent samples. All statistical analyses were conducted using GraphPad Prism 9 (version 10.0). Differences between two groups were analyzed using an independent Student's *t*‐test, while comparisons among more than two groups were performed using one‐way ANOVA. Tukey's multiple comparison test was used for further differentiation after ANOVA. Differences between groups were considered statistically significant at, ^*^ *p* < 0.05; ^**^ *p* < 0.01; ^***^ *p* < 0.001; and ^****^ *p* < 0.0001; “ns” indicated not significant.

## Conflict of Interest

The authors declare no conflict of interest.

## Supporting information



Supporting Information

## Data Availability

The data that support the findings of this study are available from the corresponding author upon reasonable request.
