# Supplementary material for: 3D‐Printed Titanium Trabecular Scaffolds with Sustained Release of Hypoxia‐Induced Exosomes for Dual‐Mimetic Bone Regeneration
Source: Adv Sci (Weinh). 2025 May 11;12(23):2500599. doi: 10.1002/advs.202500599 (PMC12199343; doi:10.1002/advs.202500599)
Supplement: Supplementary file 1 — Supporting Information [file ADVS-12-2500599-s001.docx]

**Table S1.** Primer sequences used for RT-qPCR

| Gene | Primer Sequences (F = forward; R = reverse) |
| --- | --- |
| *RUNX2* | F: TGATGAGAACTACTCCGCC |
|  | R: GTGAAACTCTTGCCTCGTC |
| *GAPDH* | F: ACTCTTCCACCTTCGATGC |
|  | R: CCGTATTCATTGTCATACCAGG |
| *ALP* | F: TTCCTGGGAGATGGTATGG |
|  | R: AATTTGTCCATCTCCAGCC |
| *OCN* | F: GGACCATCTTTCTGCTCAC |
|  | R: ACATGAAGGCTTTGTCAGAC |
| *COL1A1* | F: TGAAGAACTGGACTGTCCC |
|  | R: TTTGGTGATACGTATTCTTCCG |
| *VEGFA* | F: GATCAAACCTCACCAAAGCC |
|  | R: TCTTTCTTTGGTCTGCATTCAC |
| *CD31* | F: ACATAACAGAGCTGTTTCCCA |
|  | R: AGGACAGGTCCAACAACTC |
| *PDGFB* | F: ACTCCGTAGATGAAGATGGG |
|  | R: GAGATGAGCTTTCCAACTCG |
| *FGF2* | F: CTGCTGGCTTCTAAGTGTG |
|  | R: GAGTATTTCCGTGACCGGT |
| COL18A1 | F: CCGCGGTAGATTCTATAGGA  R: CATACCTCCCTTTATCAAGCC |
| SAMD6 | F: ACAAGCCACTGGATCTGTC  R: CTATCTGTCTGCTCACAGGG |
| PDGFA | F: GGAGATAGACTCCGTAGGG  R: TGGCTTCCTCAATACTTCTC |
| ANGPT2 | F: AGTCCAACTACAGGATTCACC |
|  | R: AATCACTTCCTGGTTGGCT |


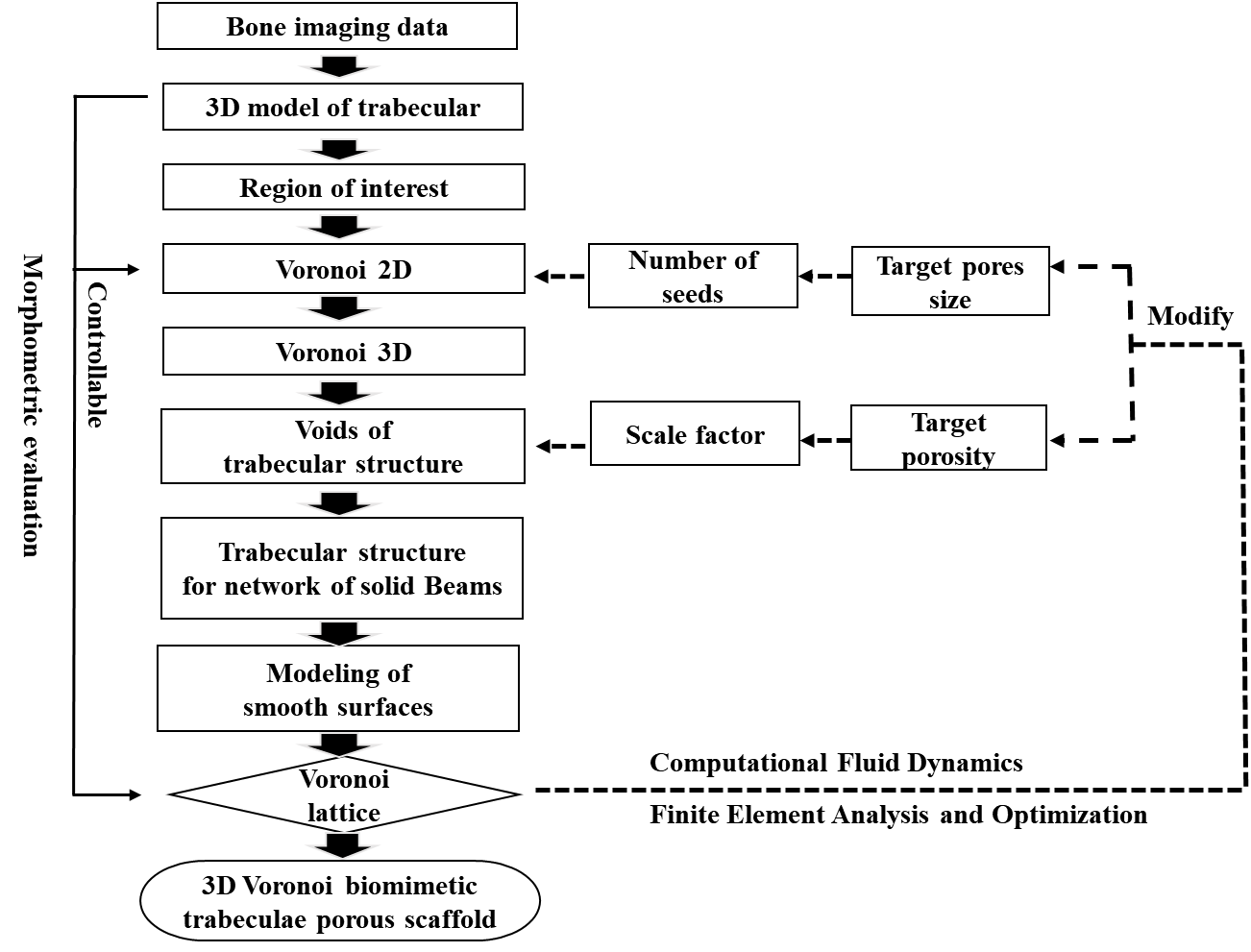


Figure S1. Schematic Workflow of Iterative Optimization for the Biomimetic Trabecular Porous Scaffold


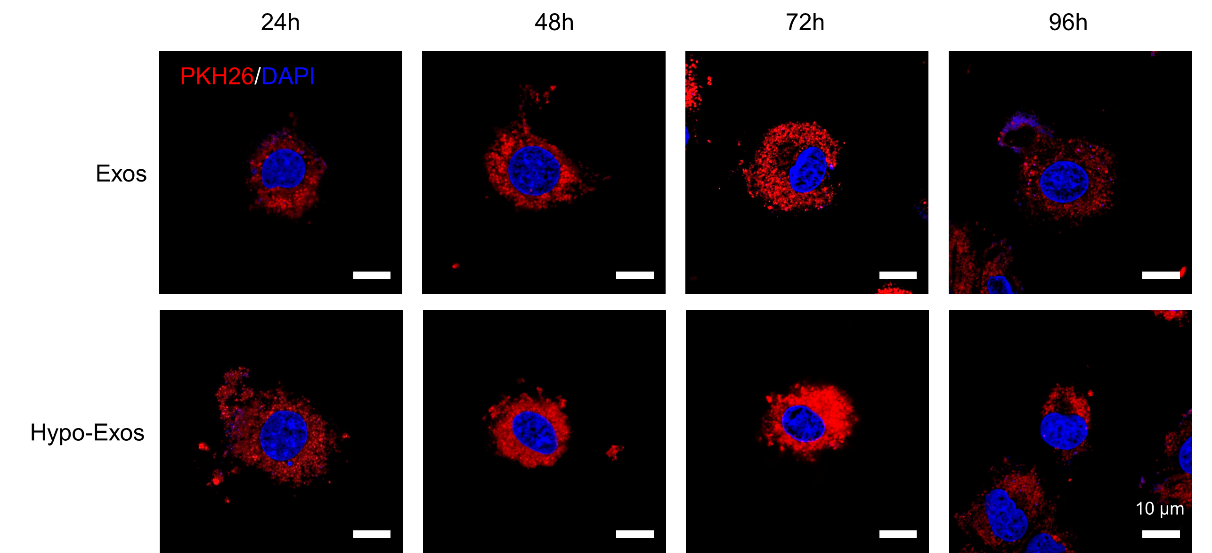


Figure S2. Fluorescence microscopy showing time-dependent internalization of PKH26-labeled exosomes (red) by recipient cells with DAPI-stained nuclei (blue), scale bar = 10 μm.


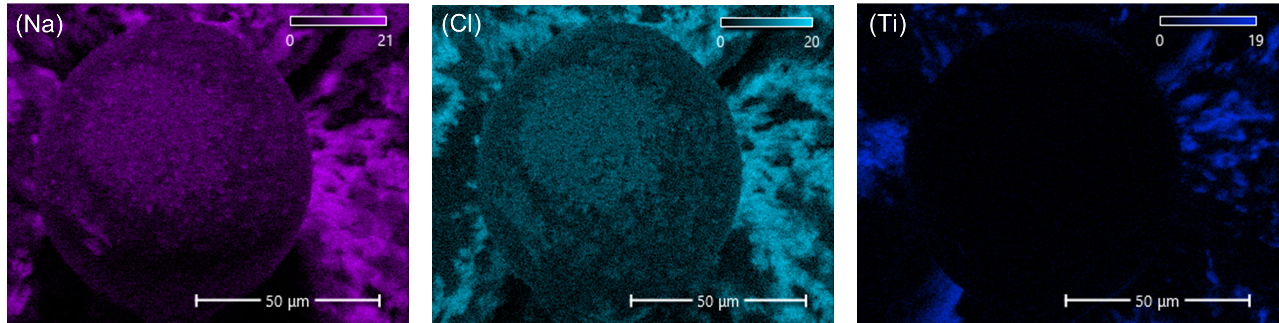


Figure S3. Elemental mapping via EDS for BTPS&pDA@PGHExo, highlighting the distribution of key elements Ti, Cl, and Na.
